# Supplementary figures and images for: Self-Organizing Properties of Mouse Pluripotent Cells Initiate Morphogenesis upon Implantation
Source: Cell. 2014 Feb 27;156(5):1032–44. doi: 10.1016/j.cell.2014.01.023 (PMC3991392; doi:10.1016/j.cell.2014.01.023)

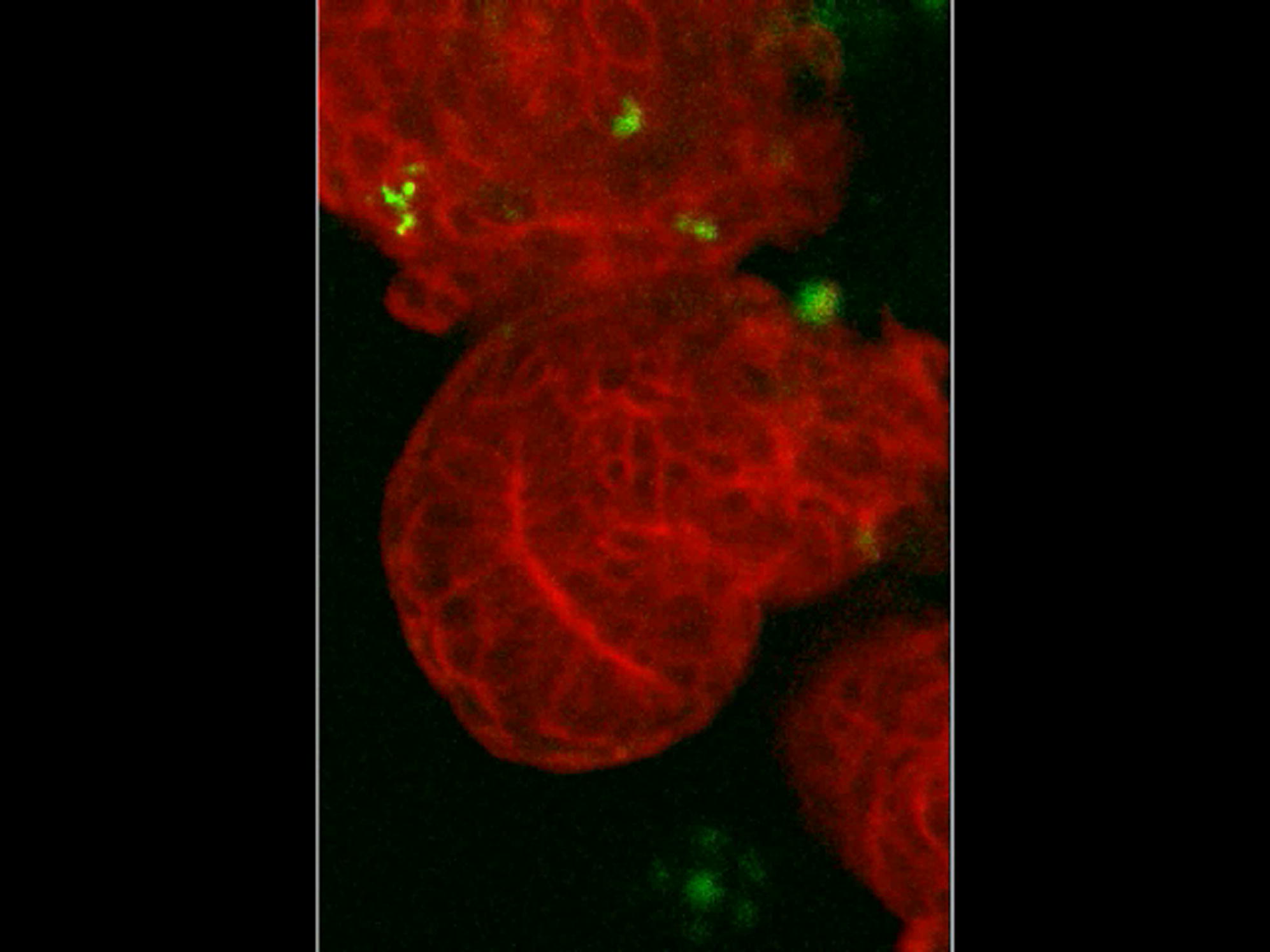

Supplement: Movie S1. Red Membrane MT/MG Embryo-Forming Egg Cylinder In Vitro, Cultured in the Presence of the Green Cell Death Reporter SYTOX, Related to Figure 1 [file mmc1.jpg]

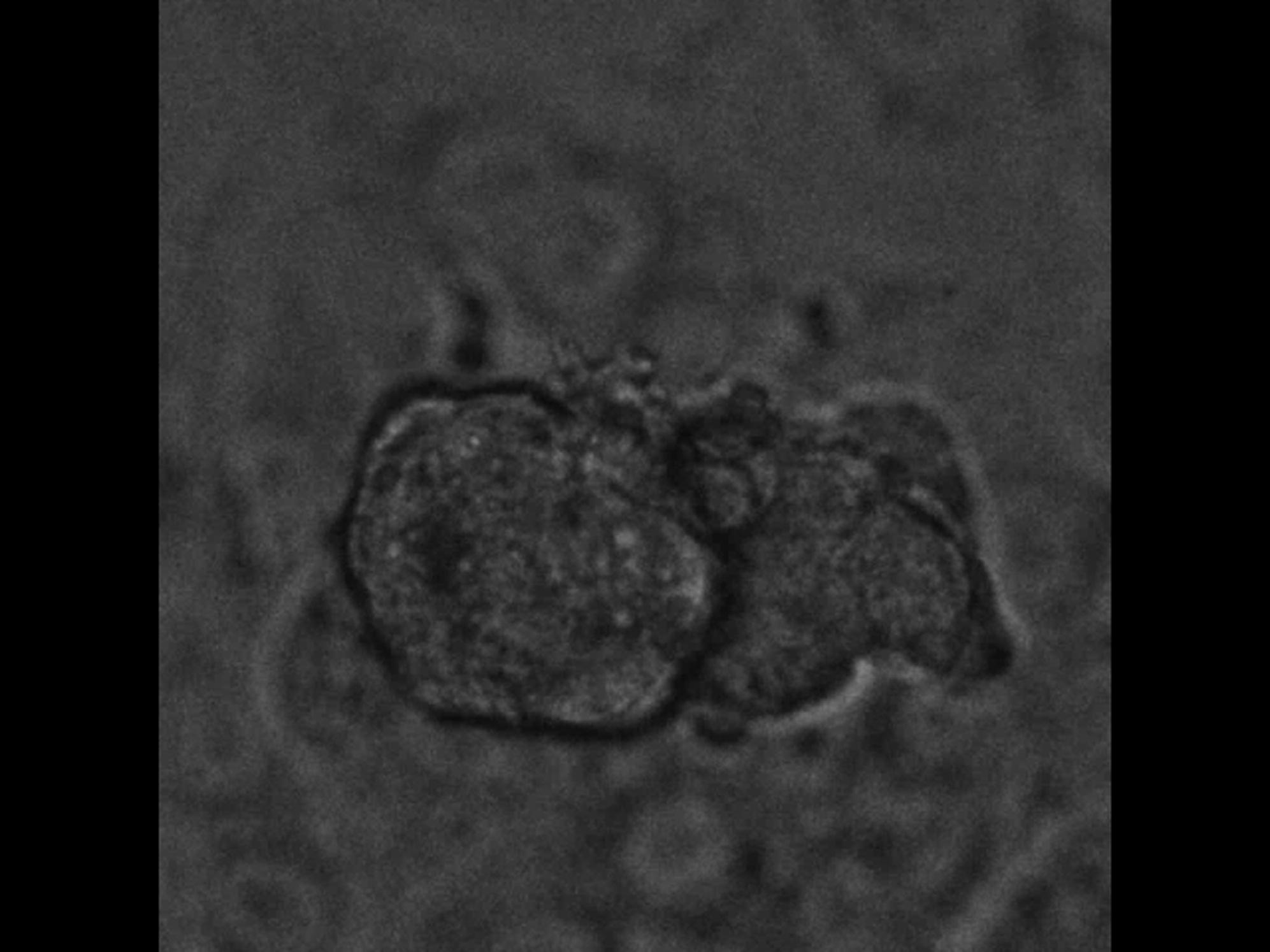

Supplement: Movie S2. Egg Cylinder Formation In Vitro in the Presence of the Green Cell Death Reporter SYTOX, Related to Figure S1 [file mmc2.jpg]

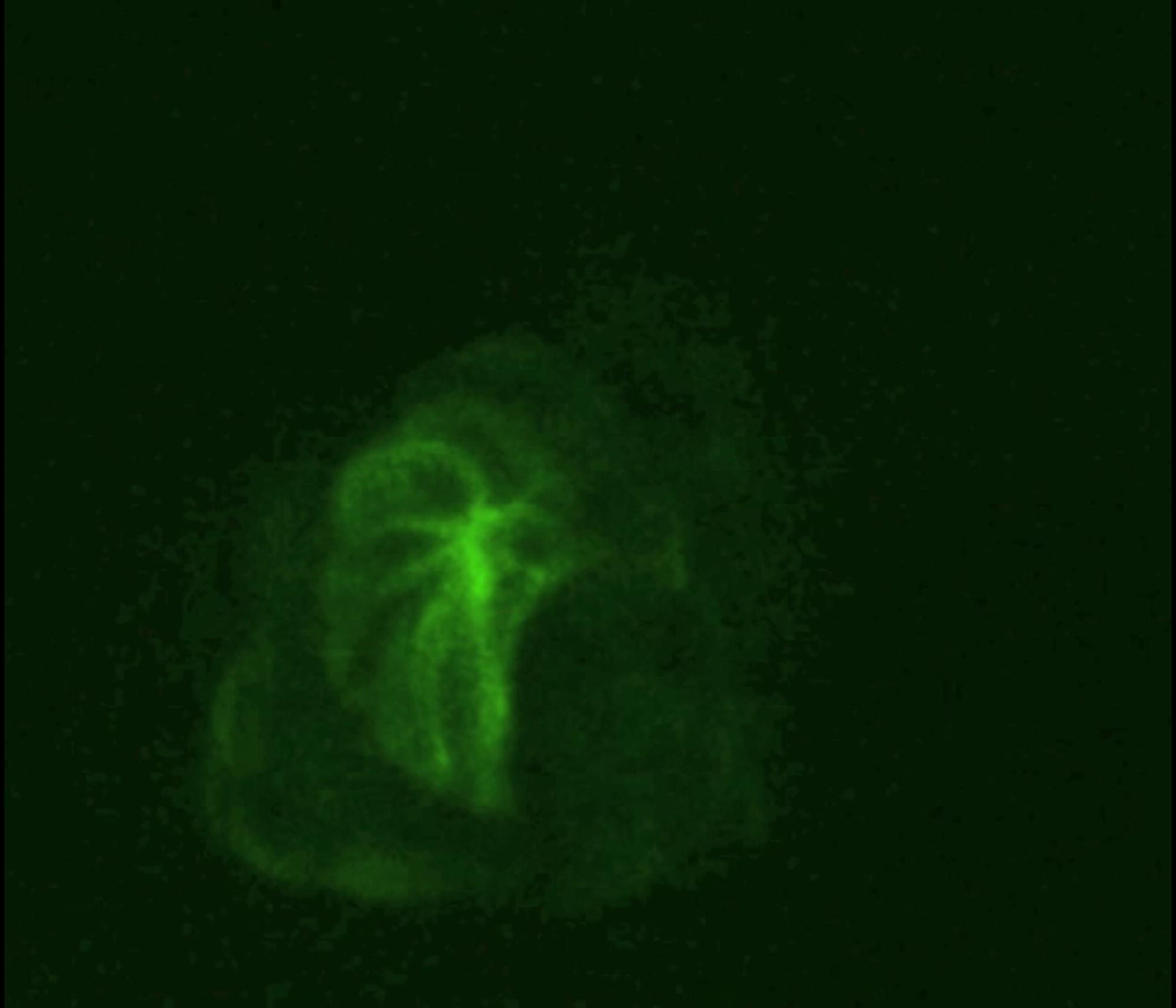

Supplement: Movie S3. Time-Lapse Recording of In-Vitro-Cultured CAG-GFP Embryo, Forming a Single Lumen from the Center of the Rosette, Related to Figure 2 [file mmc3.jpg]

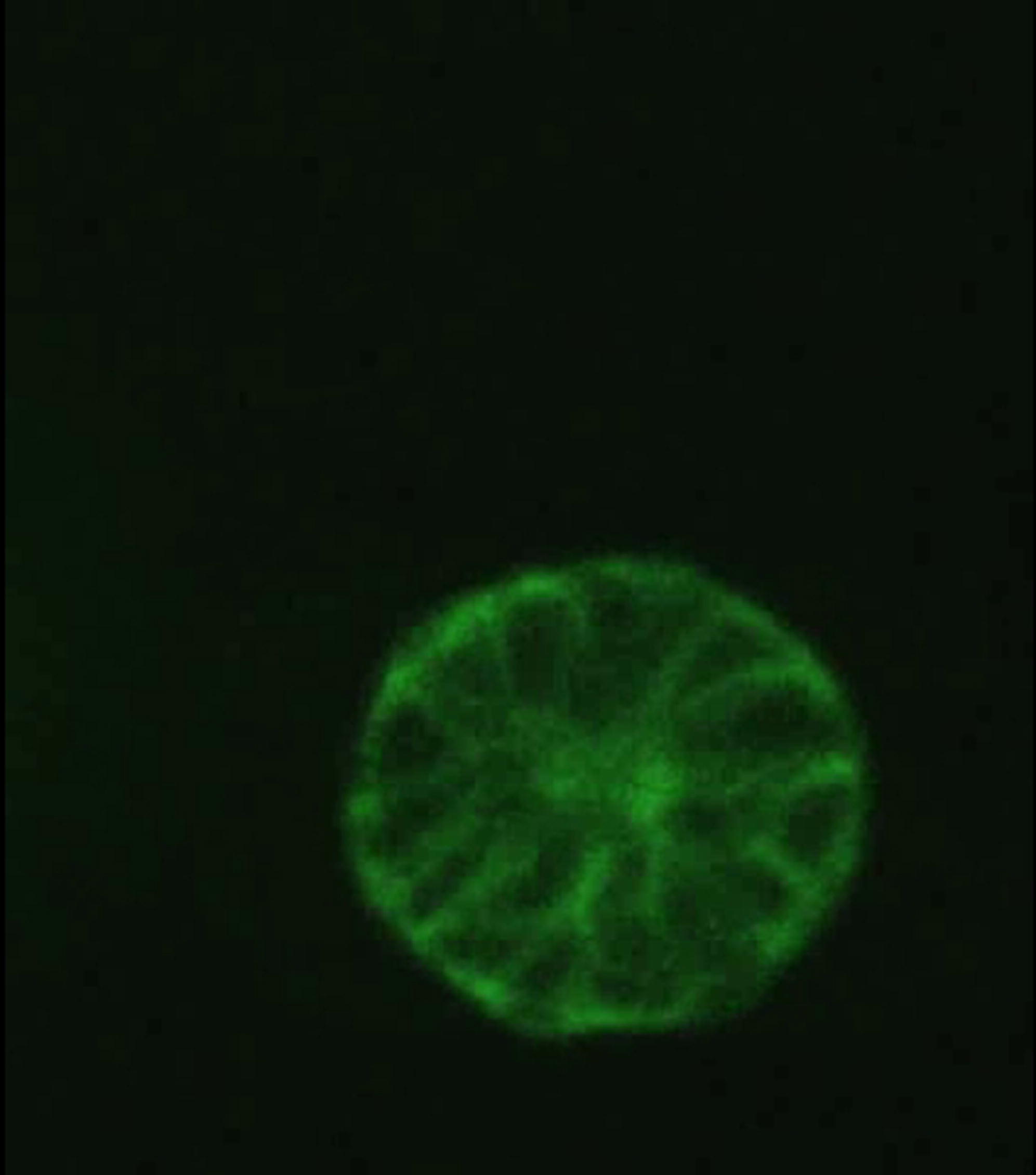

Supplement: Movie S4. Time-Lapse Recording of CAG-GFP ES Cells Embedded and Cultured in Matrigel, Related to Figure 6 [file mmc4.jpg]
