# Supplementary material for: Self-Organizing Properties of Mouse Pluripotent Cells Initiate Morphogenesis upon Implantation
Source: Cell. 2014 Feb 27;156(5):1032–44. doi: 10.1016/j.cell.2014.01.023 (PMC3991392; doi:10.1016/j.cell.2014.01.023)
Supplement: Document S1. Article plus Supplemental Information [file mmc5.pdf]

Cell

Self-Organizing Properties of Mouse Pluripotent Cells Initiate Morphogenesis upon Implantation

Ivan Bedzhov[1,2] and Magdalena Zernicka-Goetz[1,2,*]
[1]Wellcome Trust/Cancer Research UK Gurdon Institute, University of Cambridge, Tennis Court Road, Cambridge CB2 1QR, UK
[2]Department of Physiology, Development and Neuroscience, University of Cambridge, Downing Street, Cambridge CB2 3DY, UK
*Correspondence: mz205@cam.ac.uk
http://dx.doi.org/10.1016/j.cell.2014.01.023

SUMMARY

Transformation of pluripotent epiblast cells into a cup-shaped epithelium as the mouse blastocyst implants is a poorly understood and yet key developmental step. Studies of morphogenesis in embryoid bodies led to the current belief that it is programmed cell death that shapes the epiblast. However, by following embryos developing in vivo and in vitro, we demonstrate that not cell death but a previously unknown morphogenetic event transforms the amorphous epiblast into a rosette of polarized cells. This transformation requires basal membrane-stimulated integrin signaling that coordinates polarization of epiblast cells and their apical constriction, a prerequisite for lumenogenesis. We show that basal membrane function can be substituted in vitro by extracellular matrix (ECM) proteins and that ES cells can be induced to form similar polarized rosettes that initiate lumenogenesis. Together, these findings lead to a completely revised model for peri-implantation morphogenesis in which ECM triggers the self-organization of the embryo's stem cells.

INTRODUCTION

All tissues of the body originate from the pluripotent epiblast (EPI), a ball of cells positioned in the inner cell mass (ICM) of the blastocyst, whose identity is established during the first 4 days of development. During this time, the fertilized egg undergoes cleavage divisions that progressively generate three cell types. A first wave of asymmetric division at the 8–16 cell transition separates outside cells, precursors of the extra-embryonic tophectoderm (TE), from inside cells destined to be predominantly EPI (Krupa et al., 2014; Morris et al., 2013; Morris et al., 2010). Asymmetric divisions in the next cleavage rounds generate inside cells predominantly destined to become another extra-embryonic tissue, primitive endoderm (PE). When the blastocyst cavity forms, the EPI and PE are initially mixed (Chazaud et al., 2006) but then sort in an actin-dependent process to form two distinct layers (Meilhac et al., 2009; Morris et al., 2010; Plusa et al., 2008). The mature blastocyst is then ready to implant, and after hatching out of *zona pellucida*, it adheres to the uterine wall, allowing the TE to invade. This induces decidualization of the uterine stroma, burying the embryo within the maternal tissues (Dey et al., 2004). Cell proliferation and morphogenesis are initiated as the embryo implants to increase the size and to shape the tissues of the fetus.

The pre- to postimplantation transition is critical because the ensuing morphogenesis lays the foundation for the developing body to ensure developmental success. This transformation is dynamic, associated with intensive growth, and changes in the shape and topology of tissues. As a result, the initially amorphous EPI transforms into a polarized cup-shaped epithelium positioned at the distal part of the conceptus. The TE-derived extra-embryonic ectoderm (ExE) becomes positioned at the proximal part. Both EPI and ExE tissues become enveloped by PE-derived visceral endoderm (VE). The ExE and the VE will provide support for the growth of the EPI and signals for the emergence of the body plan (Tam and Loebel, 2007). In contrast to our broader understanding of the stream of events before implantation, the transition from the blastocyst into an elongated cup-like structure, the egg cylinder, remains largely unknown. This is because this morphogenesis takes place as the embryo implants and, consequently, becomes inaccessible for direct viewing and manipulations. Here, we wished to uncover the hidden sequence of events that transforms the EPI of the blastocyst into a polarized columnar epithelium within which the proamniotic cavity emerges to establish the tube of the egg cylinder.

Tubes are common units of various organs that permit a variety of functions, including gas, fluid, and ion exchange. Tubes can be formed from polarized epithelium through the wrapping of epithelial sheets, as during neural tube formation (Colas and Schoenwolf, 2001), or can be extended by budding from pre-existing tubes or sheets, as during embryonic kidney development (Hogan and Kolodziej, 2002). Depending on the dynamics of polarization, groups of cells can use two mechanisms to establish the lumen of the tube de novo: either through hollowing or by cavitation (Lubarsky and Krasnow, 2003). Hollowing, for example in the *Caenorhabditis elegans* gut formation, requires that cells become uniformly polarized to generate a lumen following the separation of their apical membranes (Leung et al., 1999). The cavitation of embryoid bodies (EBs) formed from aggregates of ES cells or embryonal carcinoma (EC) cells

 ©2014 Elsevier Inc.

is mediated by apoptosis and has become the textbook model for formation of the proamniotic cavity of the egg cylinder in the development of the mouse embryo (Coucouvanis and Martin, 1995; Wolpert, 2011). In this model, it is proposed that shortly after implantation, at embryonic day 5 (E5.0), the EPI is a solid bud surrounded by the PE-derived VE. The VE is proposed to be the source of a signal for programmed cell death in the EPI. A second signal for survival is proposed to be provided only to cells in direct contact with the surrounding basal membrane. As a result, the EPI cells in the core would undergo apoptosis to make space for the proamniotic cavity, whereas the cells contacting the basal membrane differentiate into a polarized epithelium. Thus, in the current model, it is programmed cell death that initiates the morphogenesis of the embryo at implantation stages.

While EBs present a valuable model system that recapitulates many events in the formation of the embryonic tissues, they comprise many more cells and clearly lack the organization of the blastocyst with its three distinct cell types. We therefore sought to determine the morphogenetic steps of the pre- to post-implantation EPI transition in a system more akin to the development of the embryo. To achieve this, we turned to our recently established in vitro culture (IVC) system that permits the visualization of development of the EPI and its surrounding tissues through the implantation stages (Morris et al., 2012a). The results that we present here, which are supported by a parallel analysis of embryos recovered from the mother, are strikingly different from the current concept of the pre- to postimplantation morphogenetic events. We show that the VE is not a source of apoptotic signal and that cell death is not required for the formation of the proamniotic cavity and therefore emergence of the egg cylinder. Instead, we find that, in embryos developing both in vivo and in vitro, the EPI becomes organized into a rosette-like structure of highly polarized cells and a central lumen is then formed through hollowing of their apical membranes. This is orchestrated by polarization cues from the basal membrane transmitted through β1-integrin receptors. Finally, we show that the individual or small groups of ES cells can be induced to undertake a similar process of self-organization into rosettes following their in vitro culture suspended in gels of extracellular matrix proteins. Together, our findings have uncovered a previously hidden sequence of morphogenic events and lead us to propose a complete revision of the model for the blastocyst to egg cylinder transition.

RESULTS

Programmed Cell Death Is Not Required for the Morphogenesis of the Blastocyst into Egg Cylinder

The current model of the peri-implantation morphogenesis proposes that the hollow tube of the egg cylinder is formed as a consequence of apoptosis in the core of the EPI (Coucouvanis and Martin, 1995; Wolpert, 2011). This model originally arose following observations of programmed cell death of the inner cells of EBs, leading to cavity formation. The finding that only a minority of embryos recovered from mothers at E5.0–E5.5 had apoptotic cells in the nascent cavity was then suggested to be the result of the rapid elimination of dying cells by efferocytosis (Coucouvanis and Martin, 1995; Vandivier et al., 2006). Alternatively, it could be that, although cell death is important to form cavities in EBs, it might be less so in the developing embryo. To address this required examining apoptosis in embryos as the cavity is in the process of being formed. This required a better way of modeling the time course of peri-implantation development to guide us to examine specific events in implanting embryos recovered from mothers representative of particular time frames. To this end, we turned to an in vitro culture (IVC) system that we have recently shown can capture the dynamics of embryo development through the peri-implantation stages (Morris et al., 2012a). We modified this system to enable the development of zona-freed blastocysts seeded directly onto ibiTreat microscopy-grade plastic microplates in order to facilitate time-lapse observations of development. Blastocysts were plated in the previously described medium (IVC1) (Morris et al., 2012a), but once they had attached, we changed to a modified medium (IVC2) in which we substituted human cord serum with KnockOut Serum Replacement (KSR; GIBCO) and also added β-estradiol, progesterone, N-acetyl-L-cysteine, and ITS-X (Insulin-Transferrin-Selenium-Ethanolamine) as supplements (see Experimental Procedures). Embryos developed in this modified system with comparable efficiencies to our earlier study (Morris et al., 2012a), but their development was more easily recorded by confocal microscopy, as they were grown on optical-grade plastic. Filming of blastocyst development showed that TE cells first spread over the culture surface, whereupon the EPI then began to proliferate, and morphogenesis of the egg cylinder was successfully initiated in a manner similar to that we previously described (Morris et al., 2012a). Thus, this culture system provided us with an experimental model in which we could directly determine the earliest morphogenetic events of egg cylinder formation that was far more akin to embryonic development in vivo than the model offered by EBs.

We then used confocal time-lapse microscopy to record directly the morphogenesis of cultured embryos as they transit from the blastocyst into egg cylinder. To visualize dying cells, we cultured embryos derived from a transgenic line expressing a membrane-associated red fluorescent marker (Muzumdar et al., 2007) in the presence of SYTOX, a green fluorescent reporter of cell death (Life Technologies). We observed SYTOX-positive cells in different areas as the egg cylinder first emerged and then elongated, but they were never concentrated at the site of the nascent proamniotic cavity (Figures 1A and S1A and Movies S1 and S2). In order to check whether this is also the case when embryos develop in vivo, within the mother, we isolated embryos at successive implantation stages and examined the distribution of apoptotic cells through two different methods: cleaved caspase-3 and TUNEL assays. We found that embryos recovered from mothers at E5.0–E5.5 had apoptotic cells that were distributed in all lineages and not exclusively at the center of the EPI. Moreover, we could find no evidence of apoptotic cells in a large number of morphologically normal embryos (Figures 1B, 1C, S1B, and S1D).

To functionally test whether apoptosis is required for the formation of the proamniotic cavity, we examined the development of p53-deficient embryos in which there is no p53-dependent apoptosis (Donehower, 1996). We derived embryos from p53

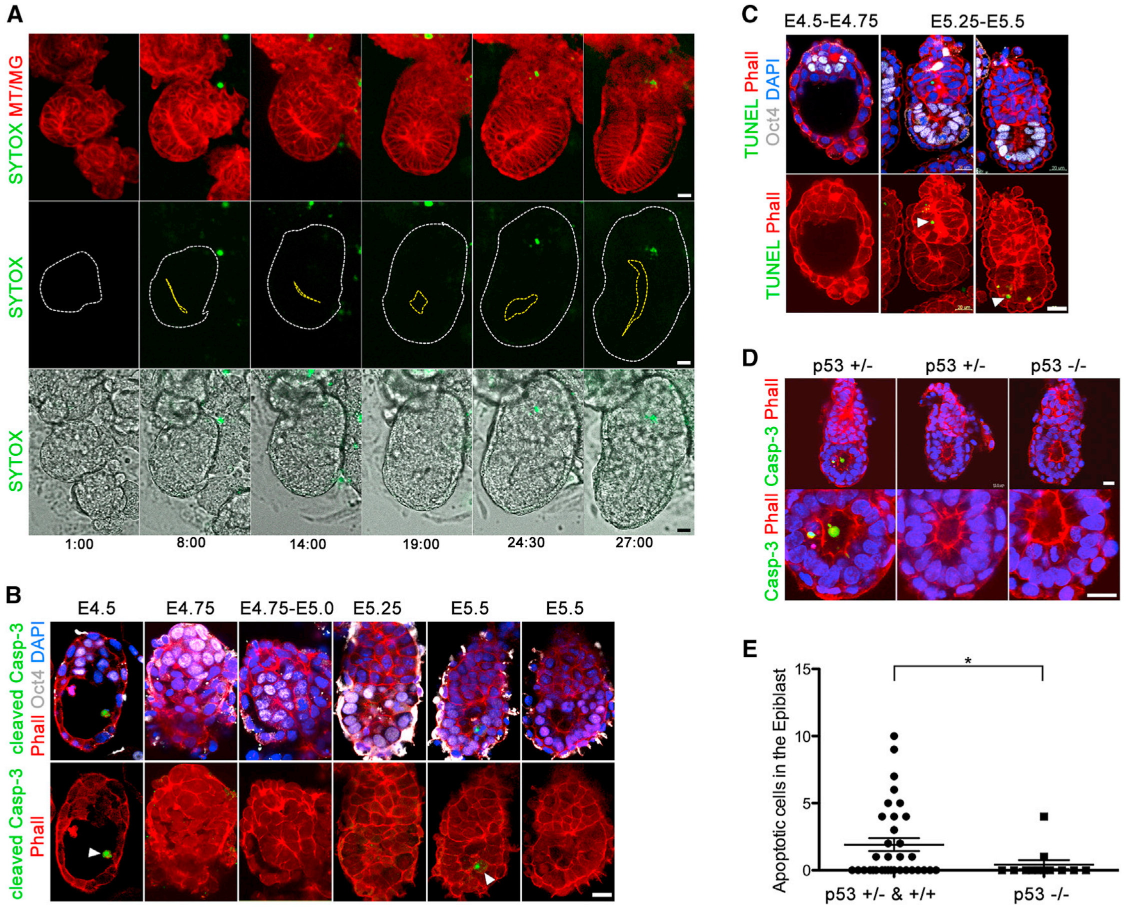

Figure 1. Cell Death in the Emerging Egg Cylinder

(A) Still images of time-lapse recording of MT/MG embryo forming egg cylinder in vitro. Dying cells are marked by SYTOX, a green cell death reporter (IVC embryos n = 23). A yellow line marks the site of the emerging proamniotic cavity.

(B) E4.5–E5.5 embryos stained for cleaved caspase-3 (arrowheads). EPI is marked by Oct4 staining (white), F-actin is labeled by phalloidin (red), and the nuclei are counterstained with DAPI (blue).

(C) Distribution of dying cells marked by TUNEL (green) at E4.5–E4.75 (n = 11 embryos) and E5.0–E5.5 (n = 27 embryos). EPI is stained for Oct4 (white), phalloidin (red), and DAPI (blue).

(D) E5.5 p53 heterozygous (+/−) and KO (−/−) embryos stained for cleaved caspase-3 (green). F-actin is labeled by phalloidin (red), and the nuclei are counterstained with DAPI (blue).

(E) Apoptotic cells in the EPI of E5.5–E6.0 embryos derived from p53 heterozygous intercrosses (p53 +/− and +/+ n = 34 embryos, p53 −/− n = 12 embryos). Almost no cleaved caspase-3-positive cells were found in the p53 KO embryos in comparison to WT and heterozygous littermates. *$p = 0.0411$, t test. Error bars represent SEM.

Scale bars, 20 μm. See also Figure S1.

heterozygous intercrosses and examined them for cleaved caspase-3-positive cells. Whereas there were variable numbers of apoptotic cells in wild-type and heterozygous embryos, such cells were completely absent from homozygous mutant p53 embryos (Figure 1D). In spite of this, the homozygous p53 mutants were morphologically indistinguishable from their wild-type and heterozygous littermates (Figures 1D and 1E). Thus, the apoptotic pathway that can be alleviated by absence of p53 appears not to be important for the morphogenesis of the egg cylinder. Because the finding that a p53 null background has no consequence for cavitation does not categorically rule out the involvement of cell death mediated by other pathways, we carried out further test of a requirement for apoptosis at this developmental stage by allowing wild-type embryos to develop in vitro in the presence of caspase-3 inhibitor. These embryos formed proamniotic cavities similar to DMSO-treated controls (Figure S1C). Thus, whether we prevent apoptosis as a result of the p53 mutation or by pharmacological treatment, the egg cylinder forms normally, strongly suggesting that cell death is not required for this process. This supports the finding of no association of cell death with the region of cavity formation. We therefore conclude that, in contrast to the current model and to what has been found in EBs, apoptosis is not essential for the formation of the proamniotic cavity, suggesting that an alternative mechanism drives morphogenesis at this stage.

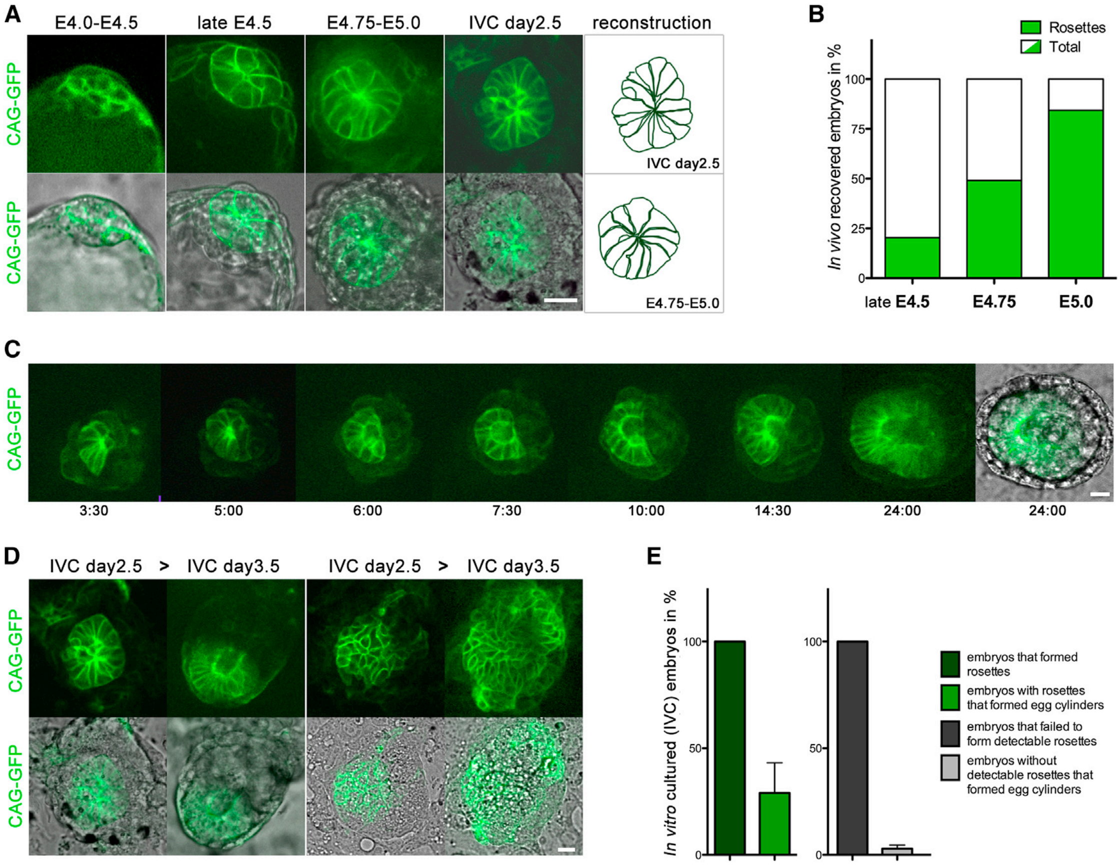

Figure 2. The Cells of the Peri-Implantation EPI Organize into a Rosette

(A) Organization of the embryonic lineage in CAG-GFP embryos revealed by high membrane GFP expression in the EPI. EPI cells change their shape from round at preimplantation (E4.0–E4.5) to wedge shaped at peri-implantation stages in vivo (late E4.5–E5.0) as well as in vitro (IVC day 2.5). The wedge-shaped cells of the EPI are arranged as a rosette.

(B) The amount of embryos with EPI organized as a rosette steadily increases from late E4.5 (20.3%, n = 11/54 embryos), E4.75 (49.1%, n = 28/57 embryos), to E5.0 (84.4%, n = 27/32 embryos).

(C) Still images of time-lapse recording of in-vitro-cultured CAG-GFP embryo. Note that a single cavity emerges from the center of the rosette.

(D) Representative snapshots of the in vitro development of individual CAG-CAG embryos. On the left, embryo with EPI rosette developed to egg cylinder. The embryo on the right failed to form a rosette and remained poorly organized.

(E) In vitro, 28% of embryos that formed rosettes developed to egg cylinders (total embryos n = 36). In contrast, only 4% of embryos without detectable rosettes gave rise to egg cylinders (total embryos n = 75), four independent experiments. Error bars represent SEM.

Scale bars, 20 μm. See also Figure S2.

EPI Is Organizing as a Rosette at the Time of Implantation

To understand exactly how the egg cylinder forms, we first focused upon the organization of the EPI at the onset of implantation. Using embryos derived from the CAG-GFP line that exhibits expression of GFP in the membrane (Rhee et al., 2006), we found that, before implantation (E4.0–E4.5), the EPI comprised round-shaped cells. As the embryo proceeded through the implantation (late E4.5–E4.75 to E4.75–E5.0), these EPI cells became wedge shaped, with their thinner parts clustering to give a rosette-like structure (Figure 2A). In embryos that have developed and hatched in utero, rosettes first appear at the late E4.5 blastocyst stage. The proportion of rosette-containing embryos then steadily increases over the next 24 hf of peri-implantation development (Figures 2B, S2A, and S2B). Such rosettes also developed in the EPI of CAG-GFP embryos cultured in vitro (Figures 2A, 2C, and 2D). As the embryo transformed into an egg cylinder, we observed a single cavity emerging at the center of the rosette (Figure 2C and Movie S3). This contrasts to the multiple cavities that appear initially at the periphery and never at the center of EBs (Coucouvanis and Martin, 1995). Importantly, we found that embryos that failed to rearrange their EPI cells into this characteristic rosette structure remained disorganized and failed to form proper egg cylinders (Figures 2D and 2E). These results indicate that the first major morphogenetic step in the transition of the EPI from pre- to post-implantation stages is its progressive reorganization from a relatively simple ball of cells to a more complex rosette-like structure that is built of polarized cells. Our results suggest that this structure is a prerequisite for proper embryonic development and acts

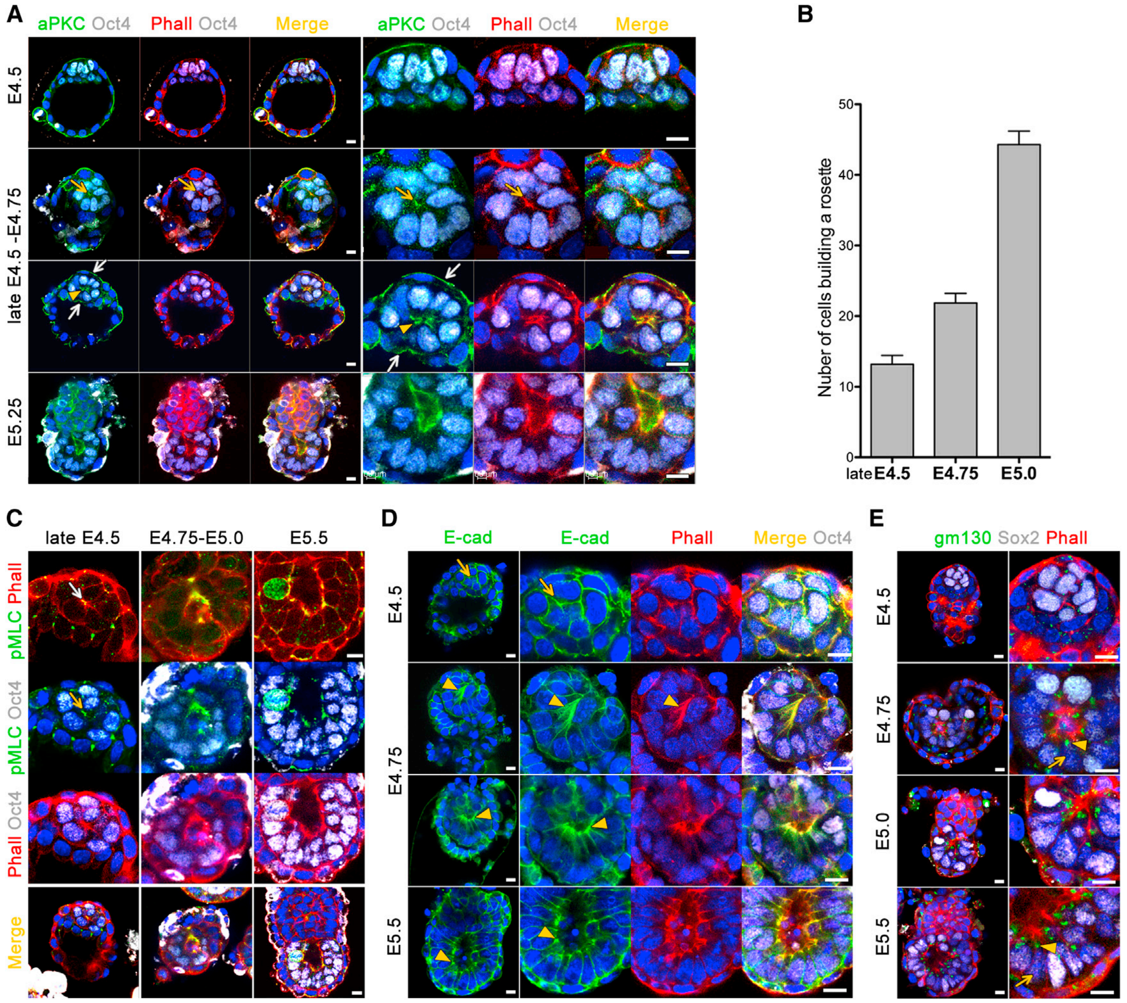

Figure 3. Establishment of Epithelial Polarity in the EPI

(A) De novo establishment of the apical domain in EPI cells. E4.5–E5.25 embryos were stained for the apical marker aPKC (green), EPI is stained for Oct4 (white), and F-actin is labeled by phalloidin (red). Low-magnification images are presented in the left, and high magnification of the EPI is in the right.

(B) The number of cells building polarized rosettes per developmental stage is steadily increasing. At late E4.5, the average cell number is 13 (n = 11 embryos), at E4.75 it is 22 (n = 35 embryos), and at E5.0 it is 44 (n = 27 embryos). Only cells expressing specific EPI markers such as Oct4, Nanog, or Sox2 were counted. Error bars represent SEM.

(C) Myosin II regulatory light-chain phosphorylation revealed by pMLC antibody staining (green). EPI is stained for Oct4 (white) and phalloidin (red).

(D) Localization of adherens junctions revealed by E-cad staining (green). EPI is labeled by Oct4 staining (white) and phalloidin (red).

(E) Localization of the Golgi apparatus is marked by gm130 antibody staining (green). The embryonic lineage is stained for Sox2 (white) and phalloidin (red). Scale bars, 10 μm. See also Figure S3.

as the foundation and organizing center of the nascent egg cylinder.

The Cells of the EPI Polarize and Constrict Apically as They Rearrange into a Rosette

Prior to implantation, the EPI comprises nonpolarized cells that become polarized during egg cylinder formation. Understanding of precisely when epithelial polarity is established in the EPI has been elusive. To address this, we followed the establishment of the apical domain marked by aPKC during epithelialization of the EPI. Expression of aPKC was not detectable in the EPI cells prior to implantation (E4.5). Then, as embryonic development progressed into the peri-implantation stages, aPKC began to accumulate centrally in the EPI together with F-actin (Figure 3A, yellow arrow). Par6 staining also confirmed that cells were becoming polarized at this stage (Figures S3A and S3B). At this time, the EPI cells rearranged into rosettes, with their apical domains clustered toward the center (Figure 3A, yellow arrowhead). As this is happening, embryos still retain blastocyst morphology and all cell lineages are polarized. Thus, the apical domains of the TE and PE face the "outside" environment and the blastocyst cavity, respectively (Figure 3A, white arrows). The apical domains of EPI cells lay within the body of the EPI, and in some cases, it was possible to detect a small lumenal

space between the apical membranes. This appeared to be the precursor of the larger proamniotic cavity of later stages (Figure 3A, yellow arrowhead).

We noted that the formation of the earliest polarized rosettes in late E4.5 embryos marked the onset of proliferation of the EPI. These early rosettes were built of an average of 12–13 cells (Figure S2). The cell number doubled around E4.75 and again around E5.0 (Figure 3B). Thus, cell number steadily increases as the embryonic development progresses through the peri-implantation stages marking the onset of cell proliferation and growth that follows the cleavage divisions of pre-implantation development.

To examine whether the shape change required for rosette formation was a consequence of specific cytoskeletal events, we examined the distribution of the phosphorylated myosin II regulatory chain. We found that myosin II regulatory chain was phosphorylated at the apical tip of the cells, where it colocalized with F-actin (Figure 3C, arrow). This would suggest that myosin II motors crosslink the actin filaments and generate contractile forces at this site. However, contractility of the actomyosin network can only affect cell shape if it is mechanically linked to the cell surface (Martin, 2010). This link is achieved in epithelial cells primarily by adherens junctions, where the cadherin-catenin complex is bound to the actin cytoskeleton. Thus, we examined the distribution of E-cadherin (E-cad), the only classical cadherin that is expressed in the EPI before gastrulation (Stemmler, 2008). We found that, whereas in pre-implantation embryos E-cad is ubiquitously distributed on the cell membrane (Figure 3D, arrow), in implanting embryos, E-cad and F-actin were localized on the apical site of the wedge-shaped cells (Figure 3D, arrowheads). Together, these results suggest that the changes in cell shape associated with EPI polarization are mediated by actomyosin-mediated constriction coupled to apical localization of the adherens junctions.

The trafficking of newly synthesized proteins from the trans-Golgi network is also essential for cell polarization, and consequently polarized epithelia show a subapical localization of Golgi apparatus and basal localization of the nucleus (Bryant and Mostov, 2008). In accord with this, we found that, whereas in pre-implantation embryos, the Golgi apparatus appeared to be distributed randomly in the cytoplasm, it became localized in the subapical region as EPI cells accumulated apical F-actin and became polarized (Figure 3E, arrowheads). At this time, the nucleus also became basally localized and the cells acquired a typical epithelial morphology (Figure 3E, arrows).

Onset of Lumen Formation in the Peri-Implantation Embryo

We then considered the mechanism behind the formation of the central lumen that takes place after acquisition of EPI polarity. In other epithelia, the lumen can be formed either by cavitation, a process driven by apoptosis, or by hollowing, the result of membrane separation. Having eliminated apoptosis as a driving force, hollowing seemed like the more likely mechanism. In podocytes and in MDCK cells, the sialomucin, podocalyxin, acts as a highly negatively charged membrane-associated anti-adhesin that maintains an open slit between neighboring cells by charge repulsion (Meder et al., 2005; Orlando et al., 2001). We therefore asked whether podocalyxin might be localized so as to provide this membrane-separating function in polarized EPI cells. We found that, before implantation, podocalyxin was expressed on the surface of the PE and later of the VE (Figure 4A, arrows). Podocalyxin then concentrated in the cytoplasm of the EPI cells at late E4.5–E4.75 (Figure 4A, arrowheads), and as the lumenal space appeared in the center of the EPI at E5.0–E5.25, podocalyxin localized on the apical surface of the cells facing the lumen (Figures 4A and 4B, arrowheads). Thus, our results suggest that the proamniotic cavity originates from a single lumen formed at the center of radially arranged and polarized EPI cells. This EPI lumen appears to be formed by hollowing, in which the apical membranes would become separated by charge repulsion likely to be mediated by anti-adhesive molecules such as podocalyxin.

We noticed that, at E5.25–E5.5, the extraembryonic ectoderm (ExE) marked by Eomes expression also developed a small intermembranous space coated by podocalyxin (Figures 4B, top, arrows and 4C, arrows). The apical domains of polarized ExE cells, marked by aPKC and F-actin, enclose this space (Figures 4D and 4E). Thus, an opening between polarized ExE cells appears to form in a similar manner to that in polarized EPI. Later at E5.75, these cavities become joined as the single lumen of the mature proamniotic cavity that spans through both the EPI and ExE (Figure 4B, bottom).

Taken together, these results suggest that the process of lumen formation in the peri-implantation embryo involves hollowing rather than apoptosis.

The Basal Membrane Creates a Niche for the Polarizing EPI

We then asked what cues within the embryo might establish EPI polarity. The orientation of epithelial polarity in MDCK cells growing in 3D culture depends on the interaction of the cells with the extracellular matrix (ECM) through integrin receptors (Yu et al., 2005), leading us to look for parallels in the mouse embryo. In pre-implantation embryos (E4.5), laminins (a major component of ECM) are secreted from TE (Figure 5A, arrow) and PE cells (Figure 5A, arrowhead) to assemble a basal membrane. At this stage and as development enters the implantation stage (E4.75), the EPI becomes enveloped by the basal membrane produced by these two extra-embryonic lineages. Later, at E5.0–E5.5, as the embryo transforms into the early egg cylinder, the basal membrane between the EPI and the ExE (derived from polar TE) is no longer maintained (Figure 5A, star). Instead, the basal membrane of the VE surrounds both the EPI and the ExE (Figure 4A, arrowheads, bottom). The β1-integrin receptor is expressed in the blastocyst before implantation and throughout the peri-implantation stages in all lineages but appeared much stronger in the region of the basal membrane (Figures 5B arrows, and 4E). This was confirmed by costaining embryos with anti-laminin to reveal the basal membrane on the basal site of the EPI cells (Figure 5C). This would be consistent with a role for the basal membrane in creating a niche for EPI cells that could sense ECM proteins through integrin receptors and thus led us to ask whether the basal membrane could provide spatial cues for polarization. To this end, we isolated the ICMs of early blastocysts by immunosurgery at a stage (E3.5) when the basal membrane is not yet assembled (Figure 5A).

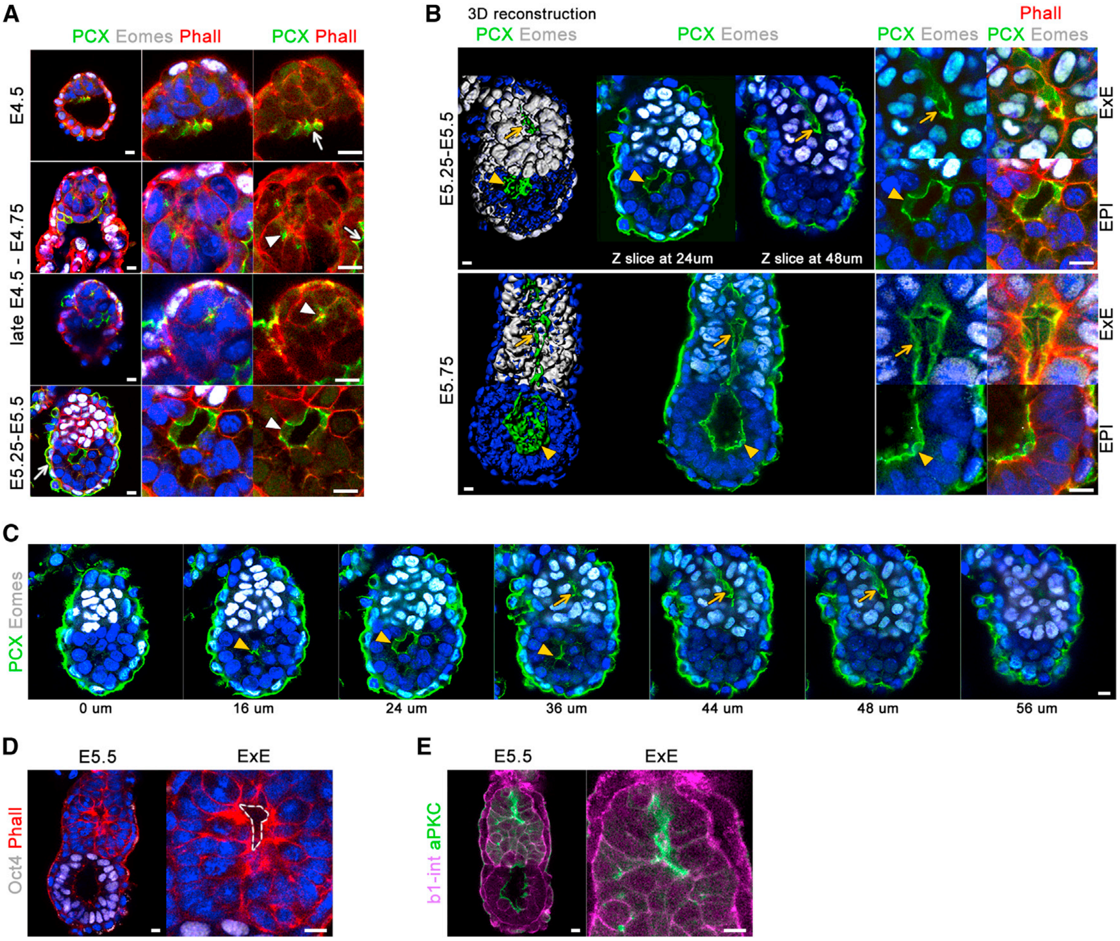

Figure 4. Lumenogenesis in the Peri-Implantation Embryo

(A) Expression and localization of podocalyxin (PCX) in early embryos (green). The TE/ExE and the VE are labeled by Eomes (white) and phalloidin (red).

(B) 3D reconstructions and optical sections of early egg cylinders stained for PCX (green) and Eomes (white). Higher magnification of the EPI and ExE regions is shown in the right, phalloidin (red) and DAPI (blue). 3D reconstruction was performed using Imaris software.

(C) Series of optical sections of E5.25–E5.5 embryo stained for podocalyxin (green) and Eomes (white). The ExE lumenal space is indicated with arrows, and the EPI lumen is indicated with arrowheads.

(D) Apical domains marked by concentrated F-actin (phalloidin, red) in ExE cells enclosing a lumen. Oct4 (white) labels the EPI.

(E) aPKC antibody staining (green) of apical domains enclosing an intermembranous slit in the EXE. β1-integrin staining is shown in magenta. Nuclei are counterstained with DAPI.

Scale bars, 10 μm.

We then embedded and cultured the ICMs in matrigel to mimic the basal membrane function in vitro (Figure 5E). Because the E3.5 ICM is a mixture of EPI and PE progenitor cells, most of the resulting structures contained Sox2-positive EPI, surrounded by Sox17-positive VE. However, about a quarter of them appeared to lack VE, and their EPI was in direct contact with the artificial ECM environment. In this latter group, the EPI became properly polarized and contained a central lumen (Figures 5D and 5F). Thus, our results indicate that the VE is not required for cavity formation and thus cannot be required to provide a death signal, as has been generally believed (Coucouvanis and Martin, 1995; Wolpert, 2011). The basal membrane niche can therefore be mimicked in vitro by the ECM molecules provided by matrigel and thus appears sufficient to support the polarization of the EPI and its maturation.

Self-Organization of ES Cells Cultured in 3D ECM

The finding that 3D culture in matrigel could provide polarization cues to EPI cells led us to ask whether this environment could provide similar cues to ES cells, which are derived from the pre-implantation EPI (Evans and Kaufman, 1981; Martin, 1981). We therefore derived ES cells from the CAG-GFP mouse line that we initially used to identify the rosettes in peri-implantation embryos in vitro and in vivo (Figure 2). We then separated CAG-GFP ES cells by brief treatment with trypsin, embedded them in matrigel, and cultured them for 48 hr (Figure 6A). Initially, the individual ES cells, together with some small clumps of two or three cells, proliferated as coherent balls. Then, between 24 and 36 hr in culture, the cells became wedge shaped, with the narrow part of the cells aligning toward the center of the structure (Figure 6A, arrowhead). Subsequently, after 36–48 hr of the culture,

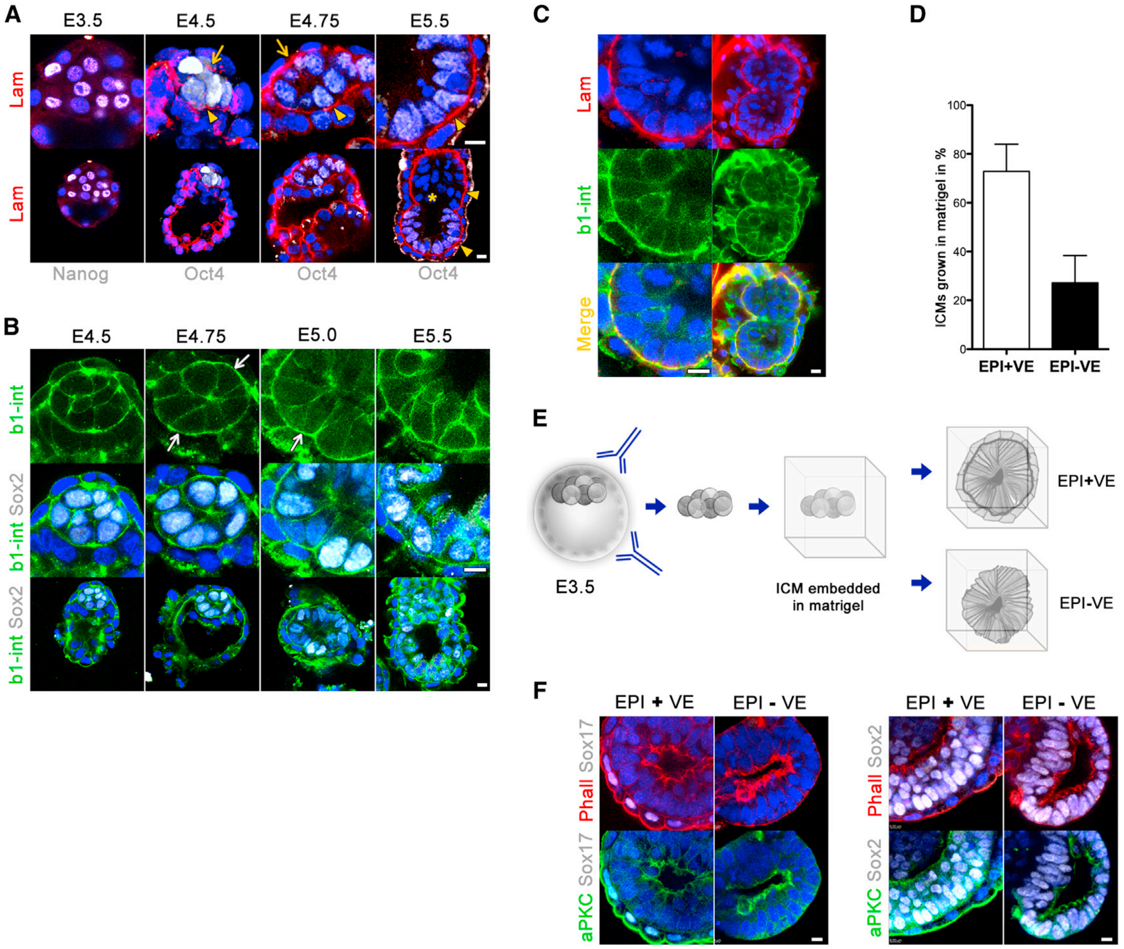

Figure 5. The Basal Membrane Creates a Niche for the Polarizing EPI

(A) TE (arrow) and PE (arrowhead) basal membranes stained for laminin (red) wrap the EPI, labeled by Oct4 or Nanog (white). As the egg cylinder emerges, the basal membrane between the ExE and the EPI is no longer maintained (star), but a common VE basal membrane surrounds both EPI and ExE (arrowheads).

(B) Expression of β1-integrin in early embryos (green); EPI is stained for Sox2 (white).

(C) Colocalization of β1-integrin (green) and laminin (red) at the basal site of the EPI.

(D) E3.5 ICMs were isolated by immunosurgery and embedded in matrigel. The majority of the EPI structures contained VE layer (72.8% in average, total ICMs n = 65), but some of the EPIs were in direct contact with the matrigel (27.1% of total ICMs n = 65); four independent experiments. Error bars represent SEM.

(E) Schematic representation of the experimental procedure.

(F) ICMs isolated by immunosurgery gave rise to polarized EPI structures surrounded by VE or directly contacting the ECM. Apical domains are marked by aPKC (green), VE is stained for Sox17 (white), and EPI is stained for Sox2 (white); phalloidin (red), DAPI (blue). Scale bars, 10 μm.

a single lumen formed in the center of these ES cell spheres (Figure 6A, arrow, and Movie S4). The organization of the cells within the sphere thus strongly resembled the EPI rosettes of the peri-implantation embryos (Figure 6B). Some cleaved caspase-3-positive cells were detected; however, their amount was not elevated in comparison to a standard 2D ES cell culture on gelatin-coated plates (Figure S4A).

To determine whether apical-basal polarity is properly established in ES cell-derived rosette structures, we cultured wild-type ES cells in matrigel and then stained them to reveal aPKC or Par6 as apical polarity markers. After 24 hr of culture, we were able to detect puncta of aPKC in the center of the rosette-like structures colocalized with F-actin (Figure 6C, arrow). By 36–48 hr of culture, ES cells were radially arranged in the spheres with their apical domains facing the lumen (Figures 6C, top, 6D, and 6E, left). We could also detect phosphorylated myosin II regulatory chain at the tip of the wedge-shaped cells colocalized with apical F-actin, as in embryo-derived EPI rosettes. Podocalyxin was also present on the apical surface of the cells surrounding the lumen just as in rosettes formed by the EPI (Figure 6E, left). Thus, apical-basal polarity is established in ES cells cultured in matrigel as the cells undergo apical constriction, and a lumen emerges between the podocalyxin-coated cell surfaces.

ES Cells Sense the ECM and Incorporate Polarity Cues through Integrin Receptors

This newly discovered ability of ES cells to form rosettes when cultured in the ECM led us to test whether ECM provides

Cell

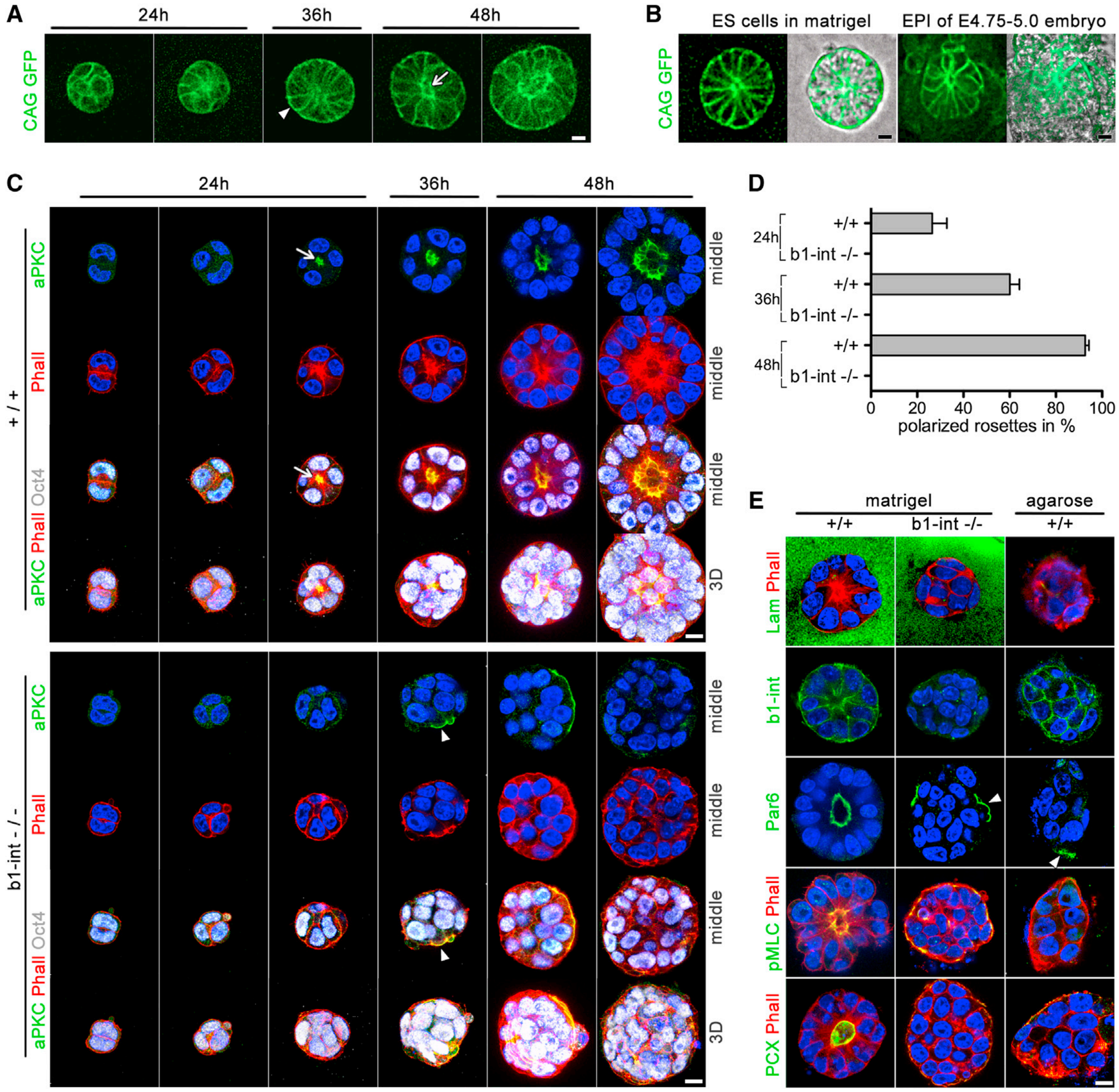

Figure 6. Self-Organization of ES Cell Spheres Grown in 3D ECM

(A) Still images of time-lapse recording of CAG-GFP ES cells' self-organization and lumenogenesis.

(B) CAG-GFP ES cells embedded in matrigel (left) compared to the peri-implantation EPI of E4.75–E5.0 CAG-GFP embryo (right).

(C) 3D projections and sections through the center of WT and β1-integrin KO ES cell spheres embedded in matrigel and stained for aPKC (green), Oct4 (white), and phalloidin (red).

(D) Formation of polarized rosettes in WT (+/+) versus β1-integrin ko (b1-int −/−) ES cells cultured in matrigel; the rate of self-organization in WT ES cell spheres was examined at 24 hr (26.5% of total spheres n = 82), 36 hr (60% of n = 195), and 48 hr (92.6% of n = 394) by aPKC or Par6 staining in > four independent experiments. β1-integrin KO ES cells failed to properly self-organize at all time points (24 hr total ES cells spheres n = 200; 36 hr n = 123; 48 hr n = 421). Error bars represent SEM.

(E) Marker analysis of WT and β1-integrin ko ES cells embedded in matrigel and WT ES cells embedded in agarose.

Scale bars, 10 μm. See also Figure S4.

polarization cues that determine proper axis of polarity, as our observations suggested. To address this, we cultured ES cells in a 3D matrix of agarose that lacked ECM proteins. We found that ES cells grew as clumps and failed to form rosettes. The clumps had some polarized cells, but these expressed Par6 on the cell membrane facing the matrix, and they failed to form a central lumen (Figures 6E, right, arrowhead, and S4B). These results strongly suggest that the ECM proteins present in the matrigel are providing cues similar to those of the basal membrane in the embryo.

The association of β1-integrin with the basal membrane led us to hypothesize that the polarization signal might be mediated by a β1-integrin-mediated signaling. In support of this, in vivo loss of function of β1-integrin leads to a failure in the EPI development at the peri-implantation stage (Fässler and Meyer, 1995; Stephens et al., 1995). To test directly the involvement of β1-integrin in

rosette formation, we followed the growth of β1-integrin −/− ES cells cultured in matrigel for 48 hr alongside control ES cells. Unlike wild-type ES cells, the β1-integrin −/− ES cells failed to self-organize into polarized rosettes but developed as clumps of round-shaped cells (Figures 6C and 6D). Some of the outer β1-integrin ES −/− cells in these clumps established apical domains, but these always faced the ECM (Figures 6C and 6E, arrowhead). The cells within these clumps remained poorly organized and failed to establish a central lumen (Figure 6E, middle). These results suggest that ES cells sense the ECM and incorporate polarity cues through integrin receptors.

Thus, our results indicate that a key event in the organization of the EPI from unpolarized into a polarized structure that occurs at the time of implantation can be mimicked by culturing ES cells suspended in a matrix of ECM proteins. The polarization and lumen formation in the resulting ES cell spheres strongly resembles the morphogenetic events during the maturation of the EPI inside the basal membrane niche and requires integrin-mediated signaling.

DISCUSSION

How the coherent ball of pluripotent cells of the blastocyst transforms into the cup-shaped monolayer of columnar epithelium enclosing the proamniotic cavity is largely a mystery because it happens while the embryo is implanting and thus hidden from view. The commonly accepted model for this process arises from studies of cavitation in EBs that have led to the textbook description of apoptosis as a major event driving the formation of this epithelium and its lumen (Coucouvanis and Martin, 1995; Wolpert, 2011). However, by studying embryos developing through these stages both in vitro, using an improved culture method, and in vivo, recovering embryos from the uterus throughout implantation, we have been able to demonstrate that apoptosis is not required in this developmental transformation. Instead, within 24 hr, the pluripotent cells polarize and constrict apically in a previously unknown morphogenetic event that reorganizes the EPI into a rosette-like structure. This apical constriction appears mediated through actomyosin contraction and transmitted through adherens junctions and is associated with the apical clustering of aPKC and Par6 and the onset of lumen formation at the center of the rosette. These events require ECM molecules of the basal membrane that is secreted by extra-embryonic lineages and that can stimulate integrin-linked signaling. We show that the role of the basal membrane can be substituted in vitro by culturing EPI or ES cells in matrigel. Together, our findings lead to revision of the model for the morphogenetic events that drive peri-implantation development (Figure 7), the component steps of which we discuss below.

We have found several important differences between the mechanisms of cavity formation in the egg cylinder and in EBs. In EBs, programmed cell death is responsible for multiple small cavities that form in their periphery. Accordingly, suppression of cell death inhibits EBs' cavitation. It was further suggested that pluripotent cells in contact with the basal membrane respond to a survival signal, enabling their differentiation into polarized columnar epithelium (Coucouvanis and Martin, 1995). When we now follow cavity formation in embryos developing either in vivo or in vitro, we find a requirement not for apoptosis but, instead, for polarization of EPI cells into a rosette-like structure. How can this difference be explained? The first important difference lies in cell number: while there are usually a few hundred in an EB on the first day of culture (Bedzhov et al., 2013; Wang and Yang, 2008), the EPI of the blastocyst comprises an average of 8–16 cells at the onset of implantation (Morris et al., 2012b). This might explain why EBs initiate formation of multiple cavities in contrast to the embryo, where only a single lumen emerges at the center of the rosette of EPI. A second important difference lies in the timing of this process: in contrast to the 24 hr required for cell polarization in the embryo, EBs need 4–7 days to establish polarized epithelium and to cavitate (Li et al., 2003). This extended time required for cell polarization may dictate an apoptotic mechanism for cavity formation. Indeed, such apoptosis-driven cavitation is also seen when canine kidney cells (MDCK) are grown at high density or in the absence of strong polarization cues and thus polarize slowly. In contrast, when MDCK cells are plated at low density and can polarize efficiently, a single central lumen forms within 2 days and independently of apoptosis. Thus, cells appear able to shift their mechanisms of de novo lumen formation, depending on the efficiency of cell polarization (Martin-Belmonte et al., 2008). In support of this, we were able to achieve efficient polarization in ES cells by culturing them at low density (individually or in small clusters) in matrigel. Such ES cells formed rosettes showing the onset of lumen formation through a hollowing mechanism within 2 days. Therefore, the timing of ES cell self-organization in 3D culture in ECM appears closer to physiological events than the cavitation of EBs.

But what triggers the key events of EPI cell polarization leading to the rosette structure and thus initiating egg cylinder formation? The results that we present here point to the importance of the basal membrane niche surrounding the EPI in providing cell polarization cues via integrin-mediated signaling. This would accord with the previously suggested role for this signaling at this developmental stage. The basal membrane comprises predominantly laminin and collagen that are secreted by the TE and PE (Li et al., 2003). Expression of laminin is directly regulated by the Sox17 transcription factor in the PE (Morris et al., 2010; Niakan et al., 2010), and PE specification, in turn, depends on Fgf4 signaling. This can account for the failure of null mutants of Fgf4 pathway components to establish PE and consequently cease development during implantation (Arman et al., 1998; Chazaud et al., 2006; Feldman et al., 1995; Frankenberg et al., 2011). Mutants for the laminin-γ1 subunit itself also fail in the peri-implantation development of the EPI, consistent with laminin's role in providing a signal for EPI reorganization (Smyth et al., 1999). The integrin-dependent sensing of the basal membrane in EPI cells accords with the failure of EPI morphogenesis and peri-implantation lethality following ablation of the β1-integrin subunit gene (Fässler and Meyer, 1995; Stephens et al., 1995). We also show that it is possible to mimic the basal membrane niche in vitro by providing ECM proteins that are sufficient to support the polarization and lumenogenesis of the EPI in the absence of VE. This leads us to propose that one essential role of the extra-embryonic lineages in peri-implantation EPI morphogenesis is to provide ECM components of the basal

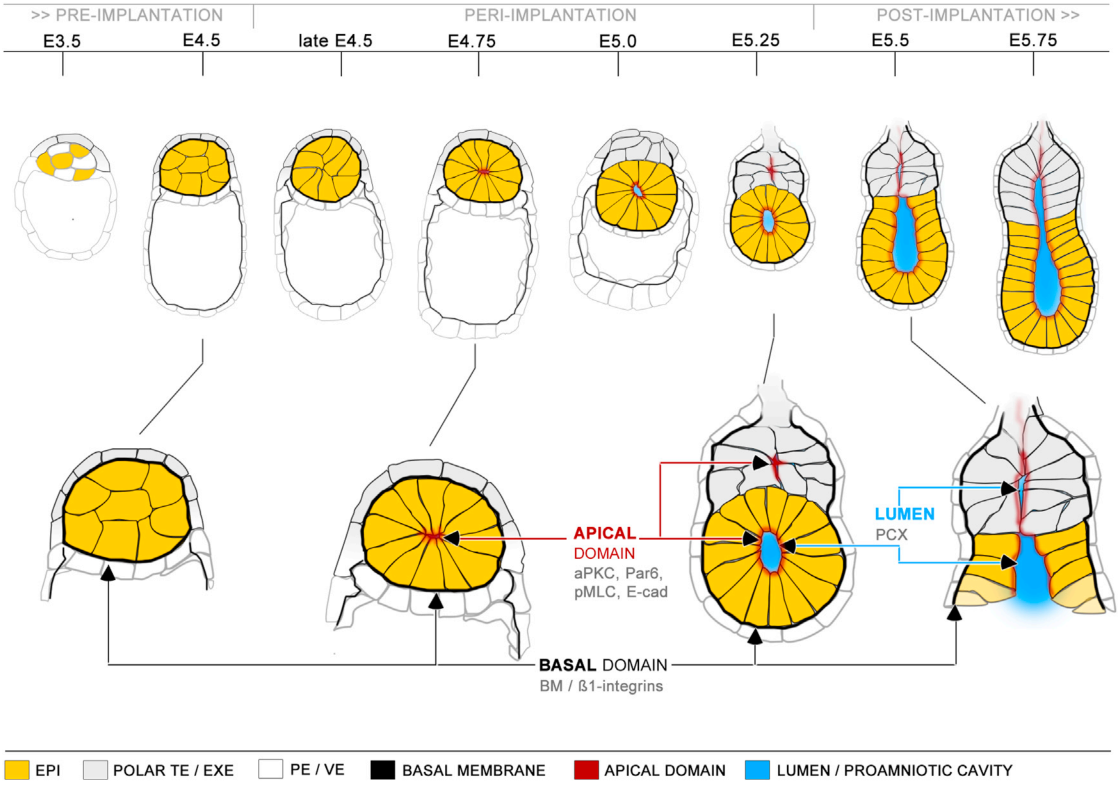

Figure 7. A Model for the Sequence of the Morphogenic Events that Drive the Peri-Implantation Development

The pre-implantation blastocyst is comprised of unpolarized EPI cells and polarized epithelia of the TE and PE (E4.5). The cells of the extraembryonic lineages secrete ECM proteins that assemble a basal membrane. The basal membrane is in direct contact on one side with the basal domain of the TE or PE cells and on the other with the EPI cells. Thereby, the basal membrane wraps around the embryonic lineage and creates a niche for its maturation during the implantation period (late E4.5–E5.25). β1-integrin receptors on the interface between the cells and the ECM enable the EPI to sense the components of the basal membrane. The ECM proteins provide polarization cues that orient the establishment of a basal-apical axis of the EPI cells. The cell membrane in direct contact with the basal membrane in the EPI periphery adopts basal identity, whereas the opposite membrane domain in the EPI center acquires apical features. Accumulation of apical determinants such as components of the Par complex establish the apical domain de novo. The cells change their shape as a result of actomyosin constriction, coupled to apical localization of adherens junctions. These radially orientated wedge-shaped cells form a rosette-like structure (E4.75–E5.0). A small lumenal space appears in the center of the rosette. The lumen is formed by hollowing, as a result of a charge repulsion of apical membranes coated by anti-adhesive glycoproteins such as podocalyxin (E5.0–E5.25). The lumen broadens as the egg cylinder elongates. Exocytosis and pumping could enhance fluid filling and could contribute to its enlargement. Similar processes of polarization and membrane repulsion generate small slits between the apical domains of ExE cells (E5.5). At that time, the EPI and the ExE are in direct contact, as the basal membrane initially covering the proximal cells of the EPI is no longer maintained. The basal membrane resembles a basket in which the basal sites of the EPI cells are anchored through integrins. Thus, the basal membrane might act as a mold to determine the global shape of the EPI, transforming it from a symmetric hollowed sphere to a cup. The lumen of the EPI cup enlarges and, together with ExE intermembranous space, forms the mature proamniotic cavity. At this point, the egg cylinder formation is complete (E5.5–E5.75).

membrane to enable EPI maturation. Importantly, our results indicate the importance of interaction of the basal membrane proteins and integrin receptors in providing spatial information that orients EPI cell polarity to enable rosette formation. This coordinated radial configuration of polarized cells enables lumenogenesis and further embryonic development.

The exact pathways downstream of basal membrane/integrin operating in the peri-implantation EPI are as yet elusive. It is not clear which of the multiple integrin-signaling pathways could lead to the Cdc42-dependent establishment of epithelial polarity that is essential for peri-implantation development (Chen et al., 2000; Wu et al., 2007). Cdc42 activates the Arp2/3 complex that leads to actin polymerization and assembly of intercellular junctions in epithelial cells; it signals to the myosin light-chain kinase and the components of the Par complex; and it is essential for proper membrane trafficking and for regulating the subapical positioning of Golgi apparatus (Etienne-Manneville, 2004). Thus, our hypothesis suggests an important role for Cdc42 to enable polarization of the EPI.

Our results suggest that the final stage of lumen formation per se requires charge repulsion between apical membranes, enabled by anti-adhesive molecules such as podocalyxin. Podocalyxin is delivered to apical membranes in other polarized cells that initiate lumen formation, including MDCK and glomerular cells (Meder et al., 2005; Orlando et al., 2001). Here, we show that podocalyxin coats the cavity of the EPI indicative of its analogous role in lumen formation. We also observe podocalyxin on the surface of ExE cells that encloses an intramembranous space, suggesting that the cavity of the ExE might form in a similar manner and that, as the egg cylinder elongates, the

lumens of the EPI and the ExE become joined. However, podocalyxin alone might not be critical for this process because of functional redundancy with other CD34 sialomucins, suggested by the postnatal lethality of podocalyxin-deficient mice (Doyonnas et al., 2001).

In summary, our findings lead to a new model for pre- to post-implantation morphogenesis (Figure 7). This model proposes that cells of the extra-embryonic lineages secrete ECM proteins that assemble a basal membrane that wraps around the embryonic lineage, creating a niche for its maturation during implantation. The ECM proteins provide polarization cues that signal through β1-integrin receptors to orient the establishment of basal-apical axis of the EPI cells. The EPI cells then change their shape as a result of actomyosin constriction countered by apically localized adherens junctions and thus form a rosette-like structure. The small lumenal space that appears in the center of the rosette is a result of charge repulsion of apical membranes coated by anti-adhesive glycoproteins such as podocalyxin. Similar processes of polarization and membrane repulsion generate small lumen between the apical domains of ExE cells that come in direct contact with the EPI because the basal membrane initially covering the proximal cells of the EPI is no longer maintained. The basal membrane resembles therefore a basket to which the basal sites of the EPI cells are anchored through integrins and might act as a mold to determine the global shape of the EPI, transforming it from a symmetric hollowed sphere to a cup.

Strikingly, the molecular mechanism controlling the pre- to postimplantation transition of the EPI can be mimicked using an in vitro model that we established using ES cells. In the presence of ECM molecules, small numbers of ES cells are able to undergo a process that resembles the polarization and lumenogenesis of the EPI in vivo. Our results indicate that, just as in embryos, basal membrane/β1-integrin signaling provides polarization cues for the coordinated orientation of the apical-basal axis of these spheres of ES cells. The cells change to a pyramidal shape, accumulating apical actin concomitantly with myosin II regulatory chain phosphorylation. The aPKC and Par6 localize on the membranes in the center of the sphere, where a single podocalyxin-coated lumen emerges. Thus, ES cells are able to undertake morphogenetic events in vitro that closely mimic the EPI transition from a ball of cell to a polarized epithelium both temporally and, most likely, mechanistically. The ability of these cells to undertake crucial self-organizing events is striking and raises the possibility that it may prove possible to carry out tests of the predictions of our new model in this simplified culture system.

EXPERIMENTAL PROCEDURES

Embryo In Vitro Culture

E3.5 embryos were recovered by flushing uteri with M2 medium. Zona pellucida were removed by brief exposure to acidic Tyrode's solution (Sigma). Zona-freed blastocysts were seeded on ibiTreat microscopy plastic μ plates (Ibidi), filled with prewarmed IVC1 medium (Advanced DMEM/F12 (GIBCO) containing 20% FCS (Biosera) and supplemented with 2 mM L-glutamine (GIBCO), 1 mM sodium pyruvate (GIBCO), penicillin (25 units/ml)/streptomycin (25 μg/ml) (GIBCO,), 1 × ITS-X (Invitrogen), 8 nM β-estradiol (Sigma), 200 ng/ml progesterone (Sigma), and 25 μM N-acetyl-L-cysteine (Sigma). In the following 36–48 hr, embryos attached to the surface of the plate as TE differentiated into giant cells. The medium was then exchanged and emerging egg cylinders cultured in serum-free, chemically defined IVC2 medium (Advanced DMEM/F12 (GIBCO) containing 30% KSR (KnockOut Serum Replacement, GIBCO) and supplemented with 2 mM L-glutamine (GIBCO), 1 mM sodium pyruvate (GIBCO), penicillin (25 units/ml)/streptomycin (25 μg/ml) (GIBCO), 1 × ITS-X (Invitrogen), 8 nM β-estradiol (Sigma), 200 ng/ml progesterone (Sigma), and 25 μM N-acetyl-L-cysteine (Sigma). Embryo culture was performed at 37°C in 5% CO_2. Procedures used for imaging living or fixed preparations of cultured embryos are given in the Extended Experimental Procedures.

ICM Immunosurgery and Culture in Matrigel

ICMs of E3.5 embryos were isolated by immunosurgery (Solter and Knowles, 1975). Individual ICMs were placed in drops of matrigel in ibiTreat microscopy plastic μ plates (Ibidi) and incubated at 37°C for 2–3 min until the matrigel solidified. The plate was then filled with warmed IVC1 medium and ICMs cultured for 3 days at 37°C in 5% CO_2.

Culture of Self-Organizing ES Cell Spheres

ES cells were washed once with PBS and were incubated for 10 min at 37°C in 0.05% trypsin-EDTA (Invitrogen). Cells were pelleted by centrifugation for 5 min/1,000 rpm, washed with PBS, and repelleted. The pellet was resuspended in matrigel (BD, 356230) by pipetting to single cells. The cell suspension was plated on ibiTreat microscopy plastic μ plates (Ibidi) and incubated for 2–3 min on 37°C until the matrigel solidified. The plate was then filled with prewarmed medium (DMEM, GIBCO) supplemented with 15% FCS (Sigma), 2 mM L-glutamine (GIBCO), 1 mM sodium pyruvate (GIBCO), penicillin 50 units/ml/streptomycin 50 μg/ml (GIBCO), 1 × NEAA (GIBCO), and 100 mM β-mercaptoethanol. Cells were cultured at 37°C and 5% CO_2. In control experiments, ES cells were cultured in 1% low melting point agarose instead of matrigel. ES cells were isolated from E3.5 CAG-GFP embryos as previously described (Stemmler and Bedzhov, 2010). β1-integrin null and control wild-type ES cells were the kind gift of Reinhard Faessler.

Animals

Animals were maintained on a 12:12 hr light-dark cycle and provided with food and water ad libitum. All experiments were in compliance with Home Office regulations. Embryos were from 4- to 6-week-old F1 mice from the C57Bl6 and CBA strains, superovulated with 10IU of pregnant mare serum gonadotropin (Intervet) and 10IU human chorionic gonadotropin (Intervet) 48 hr later and mated with F1 (C57Bl6 × CBA) or suitable transgenic male mice.

SUPPLEMENTAL INFORMATION

Supplemental Information includes Extended Experimental Procedures, four movies, and four figures and can be found with this article online at http://dx.doi.org/10.1016/j.cell.2014.01.023.

ACKNOWLEDGMENTS

We are grateful to David Glover for valuable feedback on the manuscript, Anoeska Van de Moosdijk for the contribution to the ES cell work, and to the Wellcome Trust for providing funding. β1-integrin null and control wild-type ES cells are a kind gift from Reinhard Faessler, Max Planck Institute of Biochemistry.

Received: October 17, 2013
Revised: December 24, 2013
Accepted: January 9, 2014
Published: February 13, 2014

REFERENCES

Arman, E., Haffner-Krausz, R., Chen, Y., Heath, J.K., and Lonai, P. (1998). Targeted disruption of fibroblast growth factor (FGF) receptor 2 suggests a role for FGF signaling in pregastrulation mammalian development. Proc. Natl. Acad. Sci. USA *95*, 5082–5087.

Bedzhov, I., Alotaibi, H., Basilicata, M.F., Ahlborn, K., Liszewska, E., Brabletz, T., and Stemmler, M.P. (2013). Adhesion, but not a specific cadherin code, is

indispensable for ES cell and induced pluripotency. Stem Cell Res. (Amst.) *11*, 1250–1263.

Bryant, D.M., and Mostov, K.E. (2008). From cells to organs: building polarized tissue. Nat. Rev. Mol. Cell Biol. *9*, 887–901.

Chazaud, C., Yamanaka, Y., Pawson, T., and Rossant, J. (2006). Early lineage segregation between epiblast and primitive endoderm in mouse blastocysts through the Grb2-MAPK pathway. Dev. Cell *10*, 615–624.

Chen, F., Ma, L., Parrini, M.C., Mao, X., Lopez, M., Wu, C., Marks, P.W., Davidson, L., Kwiatkowski, D.J., Kirchhausen, T., et al. (2000). Cdc42 is required for PIP(2)-induced actin polymerization and early development but not for cell viability. Curr. Biol. *10*, 758–765.

Colas, J.F., and Schoenwolf, G.C. (2001). Towards a cellular and molecular understanding of neurulation. Dev. Dyn. *221*, 117–145.

Coucouvanis, E., and Martin, G.R. (1995). Signals for death and survival: a two-step mechanism for cavitation in the vertebrate embryo. Cell *83*, 279–287.

Dey, S.K., Lim, H., Das, S.K., Reese, J., Paria, B.C., Daikoku, T., and Wang, H. (2004). Molecular cues to implantation. Endocr. Rev. *25*, 341–373.

Donehower, L.A. (1996). The p53-deficient mouse: a model for basic and applied cancer studies. Semin. Cancer Biol. *7*, 269–278.

Doyonnas, R., Kershaw, D.B., Duhme, C., Merkens, H., Chelliah, S., Graf, T., and McNagny, K.M. (2001). Anuria, omphalocele, and perinatal lethality in mice lacking the CD34-related protein podocalyxin. J. Exp. Med. *194*, 13–27.

Etienne-Manneville, S. (2004). Cdc42—the centre of polarity. J. Cell Sci. *117*, 1291–1300.

Evans, M.J., and Kaufman, M.H. (1981). Establishment in culture of pluripotential cells from mouse embryos. Nature *292*, 154–156.

Fässler, R., and Meyer, M. (1995). Consequences of lack of beta 1 integrin gene expression in mice. Genes Dev. *9*, 1896–1908.

Feldman, B., Poueymirou, W., Papaioannou, V.E., DeChiara, T.M., and Goldfarb, M. (1995). Requirement of FGF-4 for postimplantation mouse development. Science *267*, 246–249.

Frankenberg, S., Gerbe, F., Bessonnard, S., Belville, C., Pouchin, P., Bardot, O., and Chazaud, C. (2011). Primitive endoderm differentiates via a three-step mechanism involving Nanog and RTK signaling. Dev. Cell *21*, 1005–1013.

Hogan, B.L., and Kolodziej, P.A. (2002). Organogenesis: molecular mechanisms of tubulogenesis. Nat. Rev. Genet. *3*, 513–523.

Krupa, M., Mazur, E., Szczepańska, K., Filimonow, K., Maleszewski, M., and Suwińska, A. (2014). Allocation of inner cells to epiblast vs primitive endoderm in the mouse embryo is biased but not determined by the round of asymmetric divisions (8→16- and 16→32-cells). Dev. Biol. *385*, 136–148.

Leung, B., Hermann, G.J., and Priess, J.R. (1999). Organogenesis of the Caenorhabditis elegans intestine. Dev. Biol. *216*, 114–134.

Li, S., Edgar, D., Fässler, R., Wadsworth, W., and Yurchenco, P.D. (2003). The role of laminin in embryonic cell polarization and tissue organization. Dev. Cell *4*, 613–624.

Lubarsky, B., and Krasnow, M.A. (2003). Tube morphogenesis: making and shaping biological tubes. Cell *112*, 19–28.

Martin, G.R. (1981). Isolation of a pluripotent cell line from early mouse embryos cultured in medium conditioned by teratocarcinoma stem cells. Proc. Natl. Acad. Sci. USA *78*, 7634–7638.

Martin, A.C. (2010). Pulsation and stabilization: contractile forces that underlie morphogenesis. Dev. Biol. *341*, 114–125.

Martin-Belmonte, F., Yu, W., Rodriguez-Fraticelli, A.E., Ewald, A.J., Werb, Z., Alonso, M.A., and Mostov, K. (2008). Cell-polarity dynamics controls the mechanism of lumen formation in epithelial morphogenesis. Curr. Biol. *18*, 507–513.

Meder, D., Shevchenko, A., Simons, K., and Füllekrug, J. (2005). Gp135/podocalyxin and NHERF-2 participate in the formation of a preapical domain during polarization of MDCK cells. J. Cell Biol. *168*, 303–313.

Meilhac, S.M., Adams, R.J., Morris, S.A., Danckaert, A., Le Garrec, J.F., and Zernicka-Goetz, M. (2009). Active cell movements coupled to positional induction are involved in lineage segregation in the mouse blastocyst. Dev. Biol. *331*, 210–221.

Morris, S.A., Teo, R.T.Y., Li, H., Robson, P., Glover, D.M., and Zernicka-Goetz, M. (2010). Origin and formation of the first two distinct cell types of the inner cell mass in the mouse embryo. Proc. Natl. Acad. Sci. USA *107*, 6364–6369.

Morris, S.A., Grewal, S., Barrios, F., Patankar, S.N., Strauss, B., Buttery, L., Alexander, M., Shakesheff, K.M., and Zernicka-Goetz, M. (2012a). Dynamics of anterior-posterior axis formation in the developing mouse embryo. Nat. Commun. *3*, 673.

Morris, S.A., Guo, Y., and Zernicka-Goetz, M. (2012b). Developmental plasticity is bound by pluripotency and the Fgf and Wnt signaling pathways. Cell Rep. *2*, 756–765.

Morris, S.A., Graham, S.J.L., Jedrusik, A., and Zernicka-Goetz, M. (2013). The differential response to Fgf signalling in cells internalized at different times influences lineage segregation in preimplantation mouse embryos. Open Biol. *3*, 130104.

Muzumdar, M.D., Tasic, B., Miyamichi, K., Li, L., and Luo, L. (2007). A global double-fluorescent Cre reporter mouse. Genesis *45*, 593–605.

Niakan, K.K., Ji, H., Maehr, R., Vokes, S.A., Rodolfa, K.T., Sherwood, R.I., Yamaki, M., Dimos, J.T., Chen, A.E., Melton, D.A., et al. (2010). Sox17 promotes differentiation in mouse embryonic stem cells by directly regulating extraembryonic gene expression and indirectly antagonizing self-renewal. Genes Dev. *24*, 312–326.

Orlando, R.A., Takeda, T., Zak, B., Schmieder, S., Benoit, V.M., McQuistan, T., Furthmayr, H., and Farquhar, M.G. (2001). The glomerular epithelial cell anti-adhesin podocalyxin associates with the actin cytoskeleton through interactions with ezrin. J. Am. Soc. Nephrol. *12*, 1589–1598.

Plusa, B., Piliszek, A., Frankenberg, S., Artus, J., and Hadjantonakis, A.K. (2008). Distinct sequential cell behaviours direct primitive endoderm formation in the mouse blastocyst. Development *135*, 3081–3091.

Rhee, J.M., Pirity, M.K., Lackan, C.S., Long, J.Z., Kondoh, G., Takeda, J., and Hadjantonakis, A.K. (2006). In vivo imaging and differential localization of lipid-modified GFP-variant fusions in embryonic stem cells and mice. Genesis *44*, 202–218.

Smyth, N., Vatansever, H.S., Murray, P., Meyer, M., Frie, C., Paulsson, M., and Edgar, D. (1999). Absence of basement membranes after targeting the LAMC1 gene results in embryonic lethality due to failure of endoderm differentiation. J. Cell Biol. *144*, 151–160.

Solter, D., and Knowles, B.B. (1975). Immunosurgery of mouse blastocyst. Proc. Natl. Acad. Sci. USA *72*, 5099–5102.

Stemmler, M.P. (2008). Cadherins in development and cancer. Mol. Biosyst. *4*, 835–850.

Stemmler, M.P., and Bedzhov, I. (2010). A Cdh1HA knock-in allele rescues the Cdh1−/− phenotype but shows essential Cdh1 function during placentation. Dev. Dyn. *239*, 2330–2344.

Stephens, L.E., Sutherland, A.E., Klimanskaya, I.V., Andrieux, A., Meneses, J., Pedersen, R.A., and Damsky, C.H. (1995). Deletion of beta 1 integrins in mice results in inner cell mass failure and peri-implantation lethality. Genes Dev. *9*, 1883–1895.

Tam, P.P., and Loebel, D.A. (2007). Gene function in mouse embryogenesis: get set for gastrulation. Nat. Rev. Genet. *8*, 368–381.

Vandivier, R.W., Henson, P.M., and Douglas, I.S. (2006). Burying the dead: the impact of failed apoptotic cell removal (efferocytosis) on chronic inflammatory lung disease. Chest *129*, 1673–1682.

Wang, X., and Yang, P. (2008). In vitro differentiation of mouse embryonic stem (mES) cells using the hanging drop method. J. Vis. Exp. *July 23*, 825.

Wolpert, L. (2011). Principles of Development (New York: Oxford University Press).

Wu, X.W., Li, S.H., Chrostek-Grashoff, A., Czuchra, A., Meyer, H., Yurchenco, P.D., and Brakebusch, C. (2007). Cdc42 is crucial for the establishment of epithelial polarity during early mammalian development. Dev. Dyn. *236*, 2767–2778.

Yu, W., Datta, A., Leroy, P., O'Brien, L.E., Mak, G., Jou, T.S., Matlin, K.S., Mostov, K.E., and Zegers, M.M. (2005). Beta1-integrin orients epithelial polarity via Rac1 and laminin. Mol. Biol. Cell *16*, 433–445.

Supplemental Information

EXTENDED EXPERIMENTAL PROCEDURES

Time-Lapse Imaging

Confocal time-lapse imaging during in vitro culture was performed using spinning-disc microscope system (Intelligent Imaging Innovations). The embryos were imaged every 15 or 30 min in 100 μm image stacks of 8 μm z-planes. Analysis of cell death in the developing egg-cylinder was carried out using SYTOX Green nucleic acid stain (Life technologies) according to the manufacturer's instructions. Images were processed using Slidebook 5.0 (Intelligent Imaging Innovations) and Imaris software (Bitplane).

Immunofluorescence Labeling and Confocal Microscopy

Pre-implantation embryos were fixed and stained as previously described (Bedzhov et al., 2012). Peri-implantation embryos were fixed with 4% PFA/PBS for 20 min. Cellular permeabilization was carried out for 10 min in 0.1M Glycin, 0.3% Triton X-100/PBS. The embryos were in incubated in primary antibody in 10% FCS/PBT for 2h to overnight at room temperature. Secondary antibodies were applied subsequently for 2h. Embryos were stained with DAPI (Invitrogen) and mounted in PBS droplets covered with mineral oil in glass bottom petri dishes. Confocal microscopy was performed using a Leica SP5 microscope and the images were processed using Imaris software (Bitplane). Antibodies: cleaved Caspase-3 (1:200, Cell signaling, 9664), Oct4 (1:200, Santa Cruz, sc-5279), Sox2 (1:200, Santa Cruz, sc-17320), aPKC (1:200, Santa Cruz, sc-216), Par6 (1:200, Santa Cruz, sc-67393), E-cad (1:200, (Vestweber and Kemler, 1984)), gm130 (1:200, Calbiochem, CB1008), Eomes (1:200, Abcam), Nanog (1:200, Abcam, ab80892), Laminin (1:500, Sigma, L9393), Sox17 (1:200, R&D systems, AF1924), pMLC (1:200,Cell signaling, 3674), podocalyxin (1:200, R&D systems, MAB1556), β1-integrin (1:200, BD Pharmigen, 555005).

TUNEL Assay

Peri-implantation embryos were fixed with 4% PFA/PBS for 20 min. Cellular permeabilization was carried out for 10 min in 0.1M Glycin, 0.3% Triton X-100/PBS. The embryos were washed two times in PBS. TUNEL assay was performed using In Situ Cell Death Detection Kit (Roche) according to the manufacturer's instructions.

SUPPLEMENTAL REFERENCES

Bedzhov, I., Liszewska, E., Kanzler, B., and Stemmler, M.P. (2012). Igf1r signaling is indispensable for preimplantation development and is activated via a novel function of E-cadherin. PLoS Genet. *8*, e1002609.

Vestweber, D., and Kemler, R. (1984). Rabbit antiserum against a purified surface glycoprotein decompacts mouse preimplantation embryos and reacts with specific adult tissues. Exp. Cell Res. *152*, 169–178.

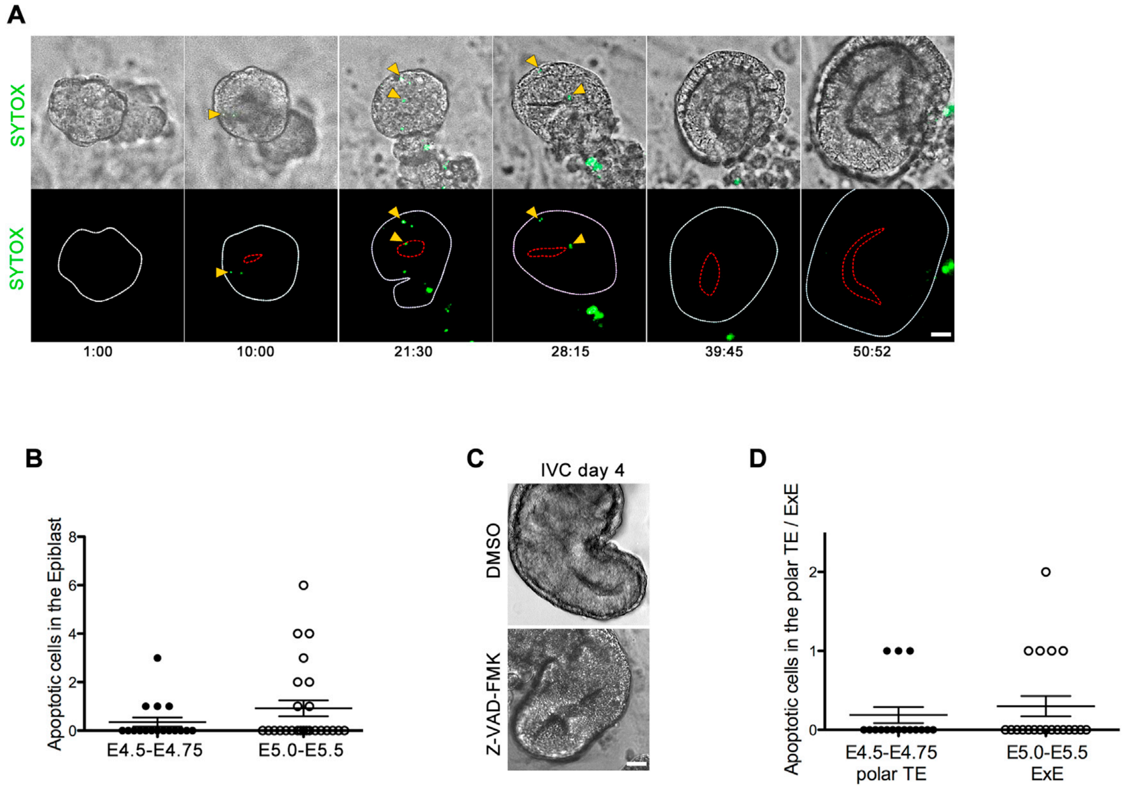

Figure S1. Cell Death during the Peri-Implantation Stages of Development, Related to Figure 1

(A) Still images of time-lapse recording of emerging egg-cylinder in vitro. Dying cells are marked by SYTOX. A red line marks the site of the proamniotic cavity.

(B) Quantification of the Cleaved Caspase-3 positive cells in the EPI of E4.5-75 (n = 17 embryos) and E5.0-5.5 embryos (n = 26 embryos). Error bars represent SEM.

(C) IVC embryos formed proamniotic cavity in the presence of 20 μM Z-DEVD FMK (n = 26 embryos), similar to DMSO treated control embryos (n = 21). Scale bar = 45 μm.

(D) Quantification of the Cleaved Caspase-3 positive cells in the polar TE of E4.5-75 (n = 16 embryos) and the ExE of E5.0-5.5 embryos (n = 20 embryos). Error bars represent SEM.

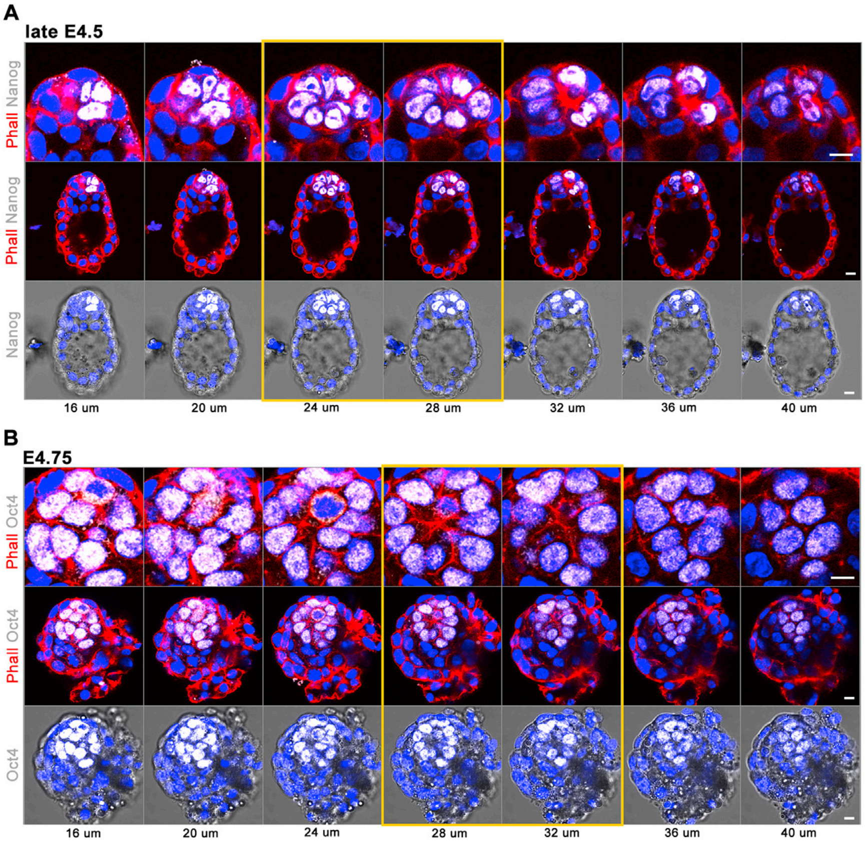

Figure S2. EPI Organizes as a Rosette in Peri-Implantation Embryos with Blastocyst Morphology, Related to Figure 2
(A and B) Optical Z-section series of immunofluorescence confocal images of late E4.5 (A) and E4.75 embryos (B). Nanog or Oct4 positive EPI cells (white) are organized as a rosette (yellow lined panels). F-actin is labeled by phalloidin (red), the nuclei are counterstained with DAPI (blue). Scale bars = 10 μm.

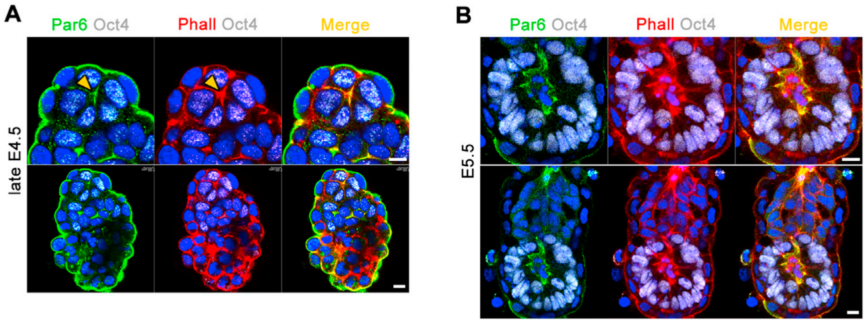

Figure S3. Polarization of the EPI during the Peri-Implantation Stages of Development, Related to Figure 3
(A and B) (A) Par6 staining (green) reveals the establishment of the apical domain in EPI cells of the late E4.5 embryo, prior to lumen formation at E5.5 (B); EPI is labeled by Oct4 (white), phalloidin (red), nuclei are counterstained with DAPI. Scale bars = 10 μm.

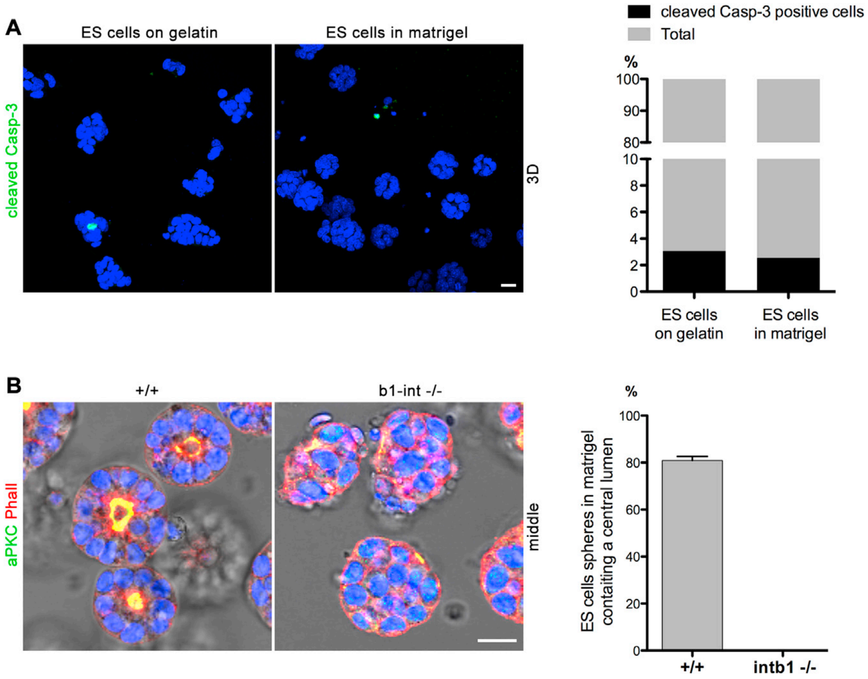

Figure S4. Apoptosis and Formation of a Central Lumen in ES Cell Spheres Grown in 3D ECM, Related to Figure 6

(A) Apoptosis in wild-type ES cell colonies grown on gelatin-coated plates and ES cell spheres cultured in matrigel for 48h. Apoptotic cells are stained for cleaved Caspase-3 (green) and the nuclei are counterstained with DAPI (blue). The proportion of cleaved Caspase-3 positive cells is similar between cells cultured on gelatin-coated plates (3%, total n = 1186 cells) versus cells cultured in 3D matrigel gels (2.5%, total n = 1012 cells).

(B) Wild-type (+/+) and β1-integrin ko (b1-int −/−) ES cells cultured in matrigel and stained for aPKC (green) and phalloidin (red). 80.7% of the wild-type ES cell spheres contained a central lumen (total n = 225 ES cell spheres) in contrast to the β1-integrin ko spheres that remained poorly organized (n = 169). Scale bars = 20 μm. Error bars represent SEM.
